# Solution structure of Z-form DNA bound to a curaxin ligand CBL0137

**DOI:** 10.1093/nar/gkag104

**Published:** 2026-02-10

**Authors:** Feifan Liu, Shiyu Wang, Yan Xu

**Affiliations:** Division of Chemistry, Department of Medical Sciences, Faculty of Medicine, University of Miyazaki, 5200 Kihara, Kiyotake, Miyazaki 889-1692, Japan; Division of Chemistry, Department of Medical Sciences, Faculty of Medicine, University of Miyazaki, 5200 Kihara, Kiyotake, Miyazaki 889-1692, Japan; Division of Chemistry, Department of Medical Sciences, Faculty of Medicine, University of Miyazaki, 5200 Kihara, Kiyotake, Miyazaki 889-1692, Japan

## Abstract

Z-DNA is known to be a left-handed alternative form of DNA and has important biological roles in cancer and other genetic diseases. In a recent study, we discovered CBL0137, a curaxin ligand, to enhance cancer immunotherapy by inducing Z-DNA formation and activating the Z-DNA-binding protein ZBP1. However, the structural information on binding complexes between Z-DNA and CBL0137 ligand has not reported to date. Here we present the first high-resolution structure of the complex between a Z-DNA and a curaxin ligand CBL0137. This compound is observed to interact with the Z-DNA through π-stacking and zig-zag localization. Furthermore, we directly observe the complex in living human cells using in-cell ^19^F NMR for the first time. This structural information provides a platform for the design of topology-specific Z-DNA-targeting compounds and is valuable for the development of new potent anticancer drugs.

## Graphical Abstract

**Graphical Abstract:**
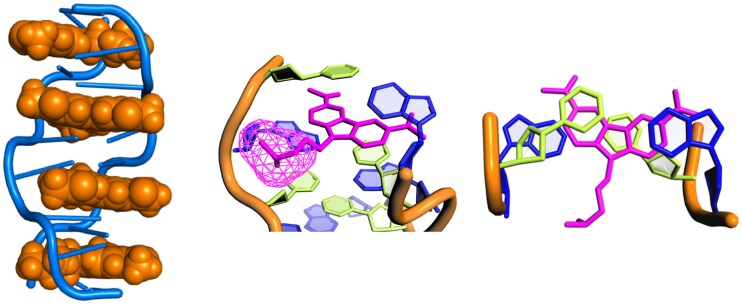

## Introduction

Z-DNA plays a critical role in gene expression [1], recombination [2, 3], and regulation [4, 5]. Recent studies have also suggested a relationship between Z-form structures and various diseases, including cancer and inflammation [6–8]. We previously reported that influenza virus-derived Z-form nucleic acids induce ZBP1-mediated necroptosis [9]. Recently, we highlighted the potential of CBL0137, a small molecule, to enhance cancer immunotherapy by inducing Z-DNA formation and activating the Z-DNA-binding protein (ZBP1) [10]. Although immune checkpoint blockade (ICB) therapy has shown success in certain cancer patients, its efficacy remains limited, partly due to tumor-intrinsic resistance mechanisms.

**Scheme 1.**
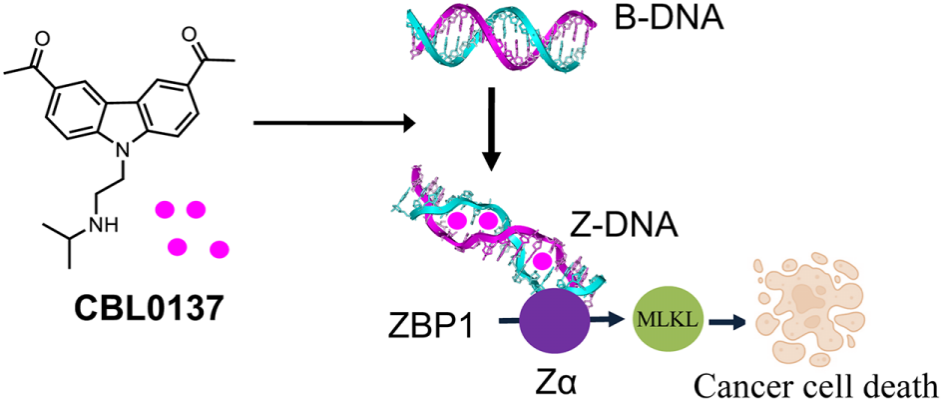
Mechanistic pathway linking CBL0137, Z-DNA, and ZBP1-mediated cell death [10]. CBL0137 promotes the B-to-Z transition of DNA, leading to the accumulation of Z-DNA that activates ZBP1 and initiates ZBP1-dependent apoptosis and necroptosis, thereby triggering cell death. This mechanism resembles the phenotype observed upon ADAR1 loss, in which the absence of ADAR1 (whose Zα domain normally binds Z-DNA) results in the accumulation of Z-DNA, activation of ZBP1, and subsequent apoptosis/necroptosis by activating MLKL protein. Such ZBP1-mediated cell death can overcome resistance to immune checkpoint blockade (ICB) therapy and enhance anti-tumor immune responses.

To address this limitation, we explored small molecules capable of inducing Z-DNA formation to activate ZBP1-dependent necroptosis of cancer cell [10]. CBL0137 promotes the B-to-Z transition of DNA, which in turn activates ZBP1-driven necroptosis (Scheme 1). One relevant mechanism involves the RNA-editing enzyme ADAR1, which suppresses type I interferon responses by preventing the accumulation of immunostimulatory double-stranded RNAs. ADAR1 contains a Zα domain that binds left-handed Z-nucleic acids (Z-DNA and Z-RNA). Loss of ADAR1 leads to the accumulation of Z-RNA and Z-DNA that activate ZBP1, triggering necroptosis and enhancing anti-tumor immunity. CBL0137-induced Z-DNA activates ZBP1 through a pathway similar to that seen with ADAR1 loss. These findings reveal that CBL0137 activates a novel immune-stimulatory pathway via Z-DNA-mediated ZBP1 activation. By mimicking the effects of ADAR1 loss, CBL0137 converts “cold” tumors into “hot” tumors, thereby improving their responsiveness to immunotherapy. Thus, targeting the ZBP1–Z-DNA axis with CBL0137 represents a promising strategy to overcome resistance to ICB therapy and enhance anti-tumor immune responses.

Despite the promising results obtained for CBL0137, the precise binding mode of this ligand to Z-DNA remains unknown, as no high-resolution structural information has been reported to date. Such information is of great importance for elucidating the underlying interaction mechanisms and for guiding the rational design and synthesis of new Z-DNA stabilizers as anticancer agents for immunotherapy.

Here, we present the first NMR solution structure of a CBL0137–Z-DNA complex. Furthermore, using in-cell ^19^F NMR spectroscopy, we directly observe this complex in living human cells for the first time. These results provide valuable insight into the structure and function of Z-DNA, and the detailed structural information will be essential for future modifications and optimization of curaxin ligands as potential anticancer drug candidates.

## Materials and methods

### DNA sample preparation

By using an automatic solid-phase phosphoramidite chemistry and DNA synthesizer, the DNAs modified with 8-trifluoromethyl-2′-deoxy-2′-fluoroguanosine analog (^8F^G) were synthesized at a scale of 1.0 μmol. The ^8F^G labelled DNA was cleaved from the column and deprotected by using AMA (Ammonium Hydroxide/40% aqueous methylamine 1:1 v/v) at room temperature for 120 min. The oligomers were further purified by high-performance liquid chromatography (HPLC) in a linear gradient of 50 mM ammonium formate in 1:1 acetonitrile/H_2_O and 50 mM ammonium formate in H_2_O. The oligomers were desalted through a NAP 10 column (GE Healthcare) and identified by MALDI-TOF-MS on an Autoflex III smart beam mass spectrometer (negative mode).

### CD measurement

CD experiments were performed by using a JASCO model J-820 CD spectrophotometer (JASCO Corporation, Tokyo, Japan). For the salt concentration experiments, the DNA sample was prepared at a 20 μM duplex concentration in the presence of 1 mM Na-PO_4_ buffer (pH 7.0) and various concentrations of sodium chloride. For the CBL0137 and DNA binding assay, DNAs were prepared at 10 μM duplex concentration in 5 mM Na-PO_4_ buffer (pH 7.0) with or without NaCl. CBL0137 was added to the DNA solution and kept at room temperature for 30 min before measurement. The binding constant *K_a_* was calculated by analyzing CD data at a fixed wavelength versus ligand concentration according to a previous study [11]. Melting temperature (T_m_) experiments were carried out using 25 μM duplex concentration in 5 mM NaPO_4_ (pH 7.0) with 1 M NaCl for modified and native DNA. The temperature was increased from 5 to 80°C at 1°C/min.

### NMR spectroscopy studying Z-form DNA structure

DNA sample, including 6 mM d(CGC^8F^GCG) (single strand concentration) with 2 M NaCl and 10 mM Na-PO_4_ buffer (pH 7.0) was prepared. Before the NMR experiment, this sample was heated to 90°C and kept for 5 min using for denaturing. Afterward, the DNA structure was renatured by decreasing the temperature of the sample to room temperature and kept overnight at 4°C. All NMR spectra were carried out using an AVANCE Bruker 500MHz spectrometer. For the exchangeable proton mode, the NMR spectra recorded in 90% H_2_O/10% D_2_O as well as water signal was suppressed using the 3–9–19 WATERGATE pulse sequence or excitation sculpting with a gradient pulse. For the non-exchangeable proton mode, the NMR spectra were recorded in 99.9% D_2_O. Mixing time was established at 300 ms, and scanning time was 360 at 20°C, used for a ^1^H NMR study. On average, 2048 complex points and 512 FIDs were collected within the spectral width of 14 097 Hz. When ^19^F NMR was studied, the ^19^F signal of CF_3_COOH (–75.66 ppm) was used as an internal standard. The data were processed with TopSpin 3.0 (Bruker BioSpinGmbh) software and analyzed with MestReNova software (12.0.1).

### NMR spectroscopy studying Z-DNA–ligand complex

1. A sample including 4 mM d(CGC^8F^GCG) (single strand concentration) with 2 M NaCl and 10 mM Na-PO_4_ buffer (pH 7.0) was prepared and heated to 90°C and kept for 5 min using for denaturing. Afterward, the temperature of this sample was decreased to room temperature and kept overnight at 4°C. Subsequently, the ligand CBL0137 was added to the above DNA solution, and got a final concentration of 4 mM. This mixture was kept at room temperature for 2 h before NMR measurement. Additionally, ligand CBL0137 alone was prepared at 4 mM with 2 M NaCl and 10 mM Na-PO_4_ buffer (pH 7.0) for the 1D NMR assay. All NMR spectra were carried out using an AVANCE Bruker 500MHz spectrometer. For the exchangeable proton mode, the NMR spectra recorded in 90% H_2_O/10% as well as water signal was suppressed using the 3–9–19 WATERGATE pulse sequence or excitation sculpting with a gradient pulse. For the non-exchangeable proton mode, the NMR spectra were recorded in 99.9% D_2_O. Mixing time was established at 200 ms, and scanning time was 360 at 20°C, used for a ^1^H NMR study. On average, 2048 complex points and 512 FIDs were collected within the spectral width of 14 097 Hz. When ^19^F NMR was studied, the ^19^F signal of CF_3_COOH (–75.66 ppm) was used as an internal standard. The data were processed with TopSpin 3.0 (Bruker BioSpinGmbh) software and analyzed with MestReNova software (12.0.1).

### Structural determination for Z-form DNA structure

All assigned NOESY cross peaks were classified as strong (1.8–3.0 Å), medium (3.0–3.7 Å), weak (3.7–5.5 Å), and very weak (5.5–7.5 Å) inter-proton distance restraints based on the intensity of NOE. The NOE peaks of H5-H6 from cytosine bases were used as calibration for the distance measurements. Distance restraints for the hydrogen bonding in each Watson-Crick base pair were 1.8–3.7 Å. The force constant of hydrogen bonds and NOE restraints was kept between 5 and 50 kcal mol^−1^ Å^−2^ throughout the computation. Then, molecular dynamics simulations were performed by the standard dynamics cascade in BIOVIA Discovery Studio 4.5 with modifications. Generally, the structure was heated from 50 K to 300 K over 4ps and equilibrated at 300 K with 100 ps simulation time. The save results interval in the production step was 2ps during a 100 ps simulation time at 300 K. The 10 best conformations generated by simulation were further energy minimized until the gradient of energy was less than 0.1 kcal mol^−1^. All conformation parameters were subjected to statistical analysis. Helical parameters, backbone, and glycosidic torsion angles for the set of 10 final structures were evaluated using the program CURVES + v.2.6 and 3DNA 2.0. Structures were displayed using PyMOL (Anaconda3; 64-bit) and BIOVIA Discovery Studio 4.5.

### Structure determination of Z-DNA–ligand complex

The solution NMR structure of Z-form d(CGC^8F^GCG)_2_ obtained in this study was used to carry out the Z-DNA–ligand docking experiment. At first, the Z-DNA model in hand was introduced into the system of BIOVIA Discovery Studio 4.5. A docking experiment using Z-form d(CGC^8F^GCG)_2_ and ligand CBL0137 was constructed by intelligent computation and provided potential docking sites used as candidates. Associating with the NOE results between DNA and ligand, appropriate cavities were determined and used for intermolecular docking. Subsequently, all NOE information, including intra- and intermolecular distance restraints in the Z–DNA–ligand complex were incorporated. These assigned NOESY cross peaks were classified as strong (1.8–3.0 Å), medium (3.0–3.7 Å), and weak (3.7–5.5 Å) inter-proton distance restraints based on the intensity of NOE. The NOE peaks of H5-H6 from cytosine bases were used as calibration for the distance measurements. Distance restraints for the hydrogen bonding in each Watson-Crick base pair were 1.8–3.7 Å. The force constant of hydrogen bonds and NOE restraints was kept between 5 and 50 kcal mol^−1 ^Å^−2^ throughout the computation. Then, molecular dynamics simulations were performed by the standard dynamics cascade in BIOVIA Discovery Studio 4.5 with modifications. Generally, the structure was heated from 50 to 300 K over 4ps and equilibrated at 300 K with 100 ps simulation time. The save results interval in the production step was 2ps during a 100 ps simulation time at 300 K. The 10 best conformations generated by simulation were further energy minimized until the gradient of energy was less than 0.1 kcal mol^−1^. All conformation parameters were subjected to statistical analysis. Helical parameters, backbone, and glycosidic torsion angles for the set of 10 final structures were evaluated using the program CURVES + v.2.6 and 3DNA 2.0. Structures were displayed using PyMOL (Anaconda3; 64-bit) and BIOVIA Discovery Studio 4.5.

### NMR-based molecular dynamics computation for DNA and complex structures

In order to construct the B-form d(CGCACGCG)/d(CGCGTGCG), NMR-based B-DNA d(CGCGCG)_2_ modeling was used (PDB: 1UQG). The 3′- and 5′-ends of double strands of DNA were extended by adding the d(CG) fragment. Next, the dG4 residue of one strand and the dC5 residue of the opposing strand were replaced by dA4 and dT5, respectively, to obtain d(CGCACGCG)/d(CGCGTGCG). NMR distance restraints of B-DNA d(CGCGCG)_2_ were used to run the molecular dynamic simulation of d(CGCACGCG)/d(CGCGTGCG), following before used method in this study. For the construction of Z-form d(CGCACGCG)/d(CGCGTGCG) and its complex with CBL0137, the NMR solution model of Z-form d(CGC^F^GCG)_2_ and its complex structure were used as initial models. Proper extension and modification were carried out. In addition, a total of four ligands were used to carry out the docking experiment when the complex was studied. NMR restraints-based molecular dynamics simulation was used to output the final model. In the case of B-form, Z-form, and complex construction of d(CGCACGCGCGCACGCG)/d(CGCGTGCGCGCGTGCG), similar methods as above were employed. Every molecular energy could be directly obtained through molecular dynamics computation using the CHARMM36 force field of BIOVIA Discovery Studio 4.5.

### Introduction of DNA and ligand CBL0137 into HeLa cells by SLO treatment

The detailed procedure could refer to our previous report [12]. HeLa cells (4 × 10^7^) grown in DMEM medium containing 10% FBS under a 5% CO_2_ atmosphere were collected and then washed twice with HBSS buffer. SLO (biologicalemia) was activated with 10 mM DTT and 0.05% BSA at 310 K for 2 h. To form pores in the plasma membrane, activated SLO was added to HeLa cells at a final concentration of 0.1 μg/ml, followed by gentle rotation incubation at 277 K for 15 min. After washing three times with ice-cold HBSS buffer, cells were incubated with 4 mM B- or Z-DNA and 4 mM ligand CBL0137 in 400 μL HBSS buffer at 296 K for 30 min, and then shaken. Cells were resealed by adding ice-cold HBSS buffer (containing 1 mM CaCl_2_). After 30 min of incubation at 277 K, the cells were washed using HBSS buffer containing 1 mM CaCl_2_ before ^19^F NMR measurement.

### ^19^F NMR experiment

For the salt concentration ^19^F NMR measurement, DNA samples of a concentration of 500 μM or 100 μM duplex were dissolved in 150 μL of the designed solution containing 1 mM Na-PO_4_ buffer (pH 7.0) and 10% D_2_O in various concentrations of NaCl. The samples were prepared by heating the ^19^F-labeled oligonucleotides at 90°C for 3 min and gradually cooling them to room temperature. The ^19^F NMR spectrum was measured on a Bruker AVANCE 600 MHz spectrometer (Bruker, Billerica, MA, USA) and referenced to the internal standard CF_3_COOH (–75.66 ppm). The experimental parameters are recorded as follows: spectral width 89.3 kHz, ^19^F excitation pulse 15.0 μs, relaxation delay 1.5 s, acquisition time 0.73 s, scan numbers 1200, line width 3, and temperature 10°C.

### In-cell ^19^F NMR measurement

DNA and ligand CBL0137-transfected cells were suspended in 200 μL of DMEM with 10% D_2_O and transferred to a Shigemi tube (Shigemi 5 mm Symmetrical NMR microtube). The experiment was performed at 296 K with a scan number value of 2048. After the intracellular NMR measurement, 100 μL of DMEM was added to the cell suspension, and the supernatant was collected by centrifugation at 4000 g for 10 min. The ^19^F NMR spectrum of the supernatant was measured with the same number of scans as the in-cell ^19^F NMR measurement. An intracellular ^19^F NMR spectrum was obtained by subtracting the ^19^F spectra of the supernatant to subtract the ^19^F signal of overview in-cell result.

## Results

### Synthesis of trifluoromethyl Z-DNA and evaluation of its interaction with CBL0137 by CD and ^19^F NMR

**Figure 1.**
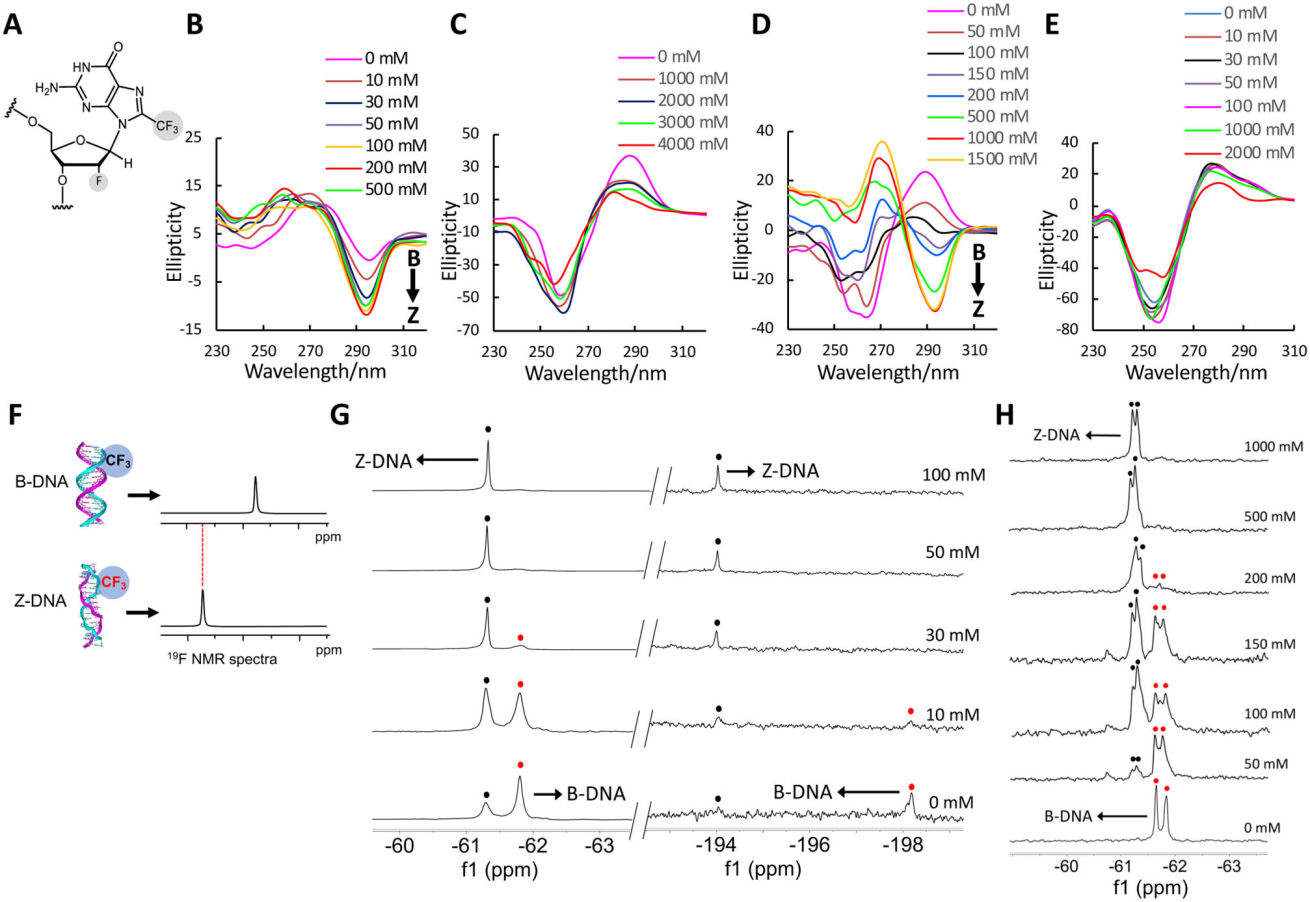
CD spectra of the B–Z transition at various NaCl concentrations and ^19^F NMR experiments for studying the B-Z transition. **(A)** Chemical structure of ^8F^G with *syn* conformation and C3′-endo sugar pucker. **(B)** CD spectra of ^8F^G modified DNA d(CGC^8F^GCG)_2_ in 1 mM Na-PO_4_ buffer (pH 7.0), at 283 K. Various NaCl concentrations are indicated. A negative Cotton effect appears at 295 nm, indicating a Z-DNA. **(C)** CD spectra of natural DNA d(CGCGCG)_2. _**(D)** CD spectra of ^8F^G modified DNA d(C^8F^GCAC^8F^GCG)/d(CGCGTGCG). **(E)** CD spectra of natural DNA d(CGCACGCG)/d(CGCGTGCG). **(F)** Concept for the detection of B–Z transition by ^19^F NMR. Two ^19^F resonances of different chemical shifts are expected according to B-DNA and Z-DNA. **(G)**  ^19^F NMR spectra of d(CGC^8F^GCG)_2_ in 1 mM Na-PO_4_ buffer (pH 7.0) and various NaCl concentrations. The ^19^F NMR spectra were recorded at 0.5 mM duplex concentration. Red and black spots indicated B-form and Z-form DNA, respectively.**(H)**  ^19^F NMR spectra of d(C^8F^GCAC^8F^GCG)/d(CGCGTGCG) in 1 mM Na-PO_4_ buffer (pH 7.0) and various NaCl concentrations.

To obtain the solution structure of Z-form DNA bound to CBL0137, we performed site-specific incorporation of the 8-trifluoromethyl-2′-deoxy-2′-fluoroguanosine analog (^8F^G) into DNA sequences using phosphoramidite chemistry (Fig. 1A, Supplementary Scheme S1). We have previously demonstrated that methylation and trifluoromethyl substitution at the C8 position of guanosine promote the *syn* conformation [12, 13], thereby stabilizing the Z-form. In the present study, additional fluorine modification at the 2′ position of the sugar moiety was introduced to further stabilize the Z-form by favoring the C3′-endo sugar pucker.

Circular dichroism (CD) spectroscopy is used to distinguish the B- and Z-form DNA [13]. For Z-DNA, a negative cotton effect is located at 295 nm, while in B-DNA, a predominant band appears at 280 nm. This difference allows us to monitor the DNA structural conversion with varying NaCl concentrations by using CD spectroscopy (Fig. 1B–E). We observed that the 6-mer duplex d(CGC^8F^GCG)_2_ with one ^8F^G remarkably stabilized the Z-DNA conformation, displaying a characteristic Z-type CD spectrum even at low NaCl concentrations (Fig. 1B). The B–Z transition midpoint was 14 mM NaCl (Table 1). In contrast, the natural sequence d(CGCGCG)_2_ did not readily undergo B–Z transition, with a midpoint at 2600 mM NaCl (Fig. 1C, Table 1). The Z-DNA stabilizing effect was further confirmed in the 8-mer duplex d(C^8F^GCAC^8F^GCG)/d(CGCGTGCG), which contains an AT base pair that normally disfavors Z-DNA formation. The duplex containing two ^8F^G residues exhibited a B–Z transition midpoint at 126 mM NaCl, significantly lower than that of the natural 8-mer duplex (>5000 mM) (Fig. 1D and E, Table 1). These results collectively suggested that the ^8F^G can dramatically stabilize the Z-DNA conformation at a physiological salt condition. We incorporated 2′F-G into the 8-mer DNA sequence *d*(C^2F^GCAC^2F^GCG)/d(CGCGTGCG). CD analysis revealed a characteristic negative band at 295 nm at high NaCl concentrations, indicating stabilization of the Z-DNA conformation (Supplementary Fig. S1). The midpoint of the B–Z transition was determined to be 1550 mM NaCl (Table 1), which is lower than that of the non-modified native 8-mer DNA sequence d(CGCACGCG)/d(CGCGTGCG). These results are consistent with previous reports demonstrating that 2′-OMe or 2′F modifications stabilize Z-DNA by adopting a C3′-endo sugar conformation that favors the Z-form DNA structure [14, 15].

**Table 1.** Midpoint NaCl concentrations for the B–Z transition in ^8F^G modified DNA sequences

Oligonucleotides	NaCl (mM)
d(CGCGCG)_2_	2600
d(CGC^8F^GCG)_2_	14
d(CGCACGCG)/d(CGCGTGCG)	>5000
d(C^8F^GCAC^8F^GCG)/d(CGCGTGCG)	126
d(C^2F^GCAC^2F^GCG)/d(CGCGTGCG)	1550

We examined the melting temperature (T_m_) using CD melting experiments. The ^8F^G-incorporated oligonucleotide exhibited a significantly higher T_m_ value (39.3°C) compared with the native DNA (26.0°C) (Supplementary Fig. S2). This observation is consistent with our previous study showing that C8- and 2′-substituted DNA exhibits enhanced thermal stability [14]. This enhanced stability can be attributed to the 8-trifluoromethyl and 2′-fluorine modifications, which favor the *syn* conformation of the base and the C3′-endo sugar pucker, thereby enhancing the thermodynamic stability of Z-DNA.

Recently, we have successfully employed ^19^F NMR to monitor various nucleic acid conformations with critical biological functions in cells, leveraging the sensitivity and chemical shift dispersion of ^19^F nuclei to distinguish biomolecular structures in complex physiological environments [16–18]. Importantly, the distinct ^19^F chemical shifts of B- and Z-DNA enable clear differentiation of the two forms in ^19^F NMR spectra (Fig. 1F).

In 6-mer duplex DNA d(CGC^8F^GCG)_2_, the major fluorine signal of the CF_3_ group was observed at − 61.8 ppm with a minor signal at − 61.3 ppm in the absence of NaCl, while that of the 2′F group appeared at − 198.2 ppm (Fig. 1G). The intensity of the minor signal is markedly greater than that of the initial peak as the NaCl concentration increases, while the major signal disappears, indicating that the Z-form DNA for the minor signal at − 61.3 ppm and the B-form DNA for the major signal were consistent with the CD result. The new peak of the 2′F group at − 194.1 ppm is assigned as a Z-DNA. In an 8-mer duplex, two ^19^F NMR peaks result from two asymmetric ^8F^G due to their different positions within the duplex sequence (Fig. 1H). With increasing NaCl concentration, the two peaks significantly decreased and completely disappeared in B-DNA, and two new strong-intensity peaks appeared as Z-DNA.

**Figure 2.**
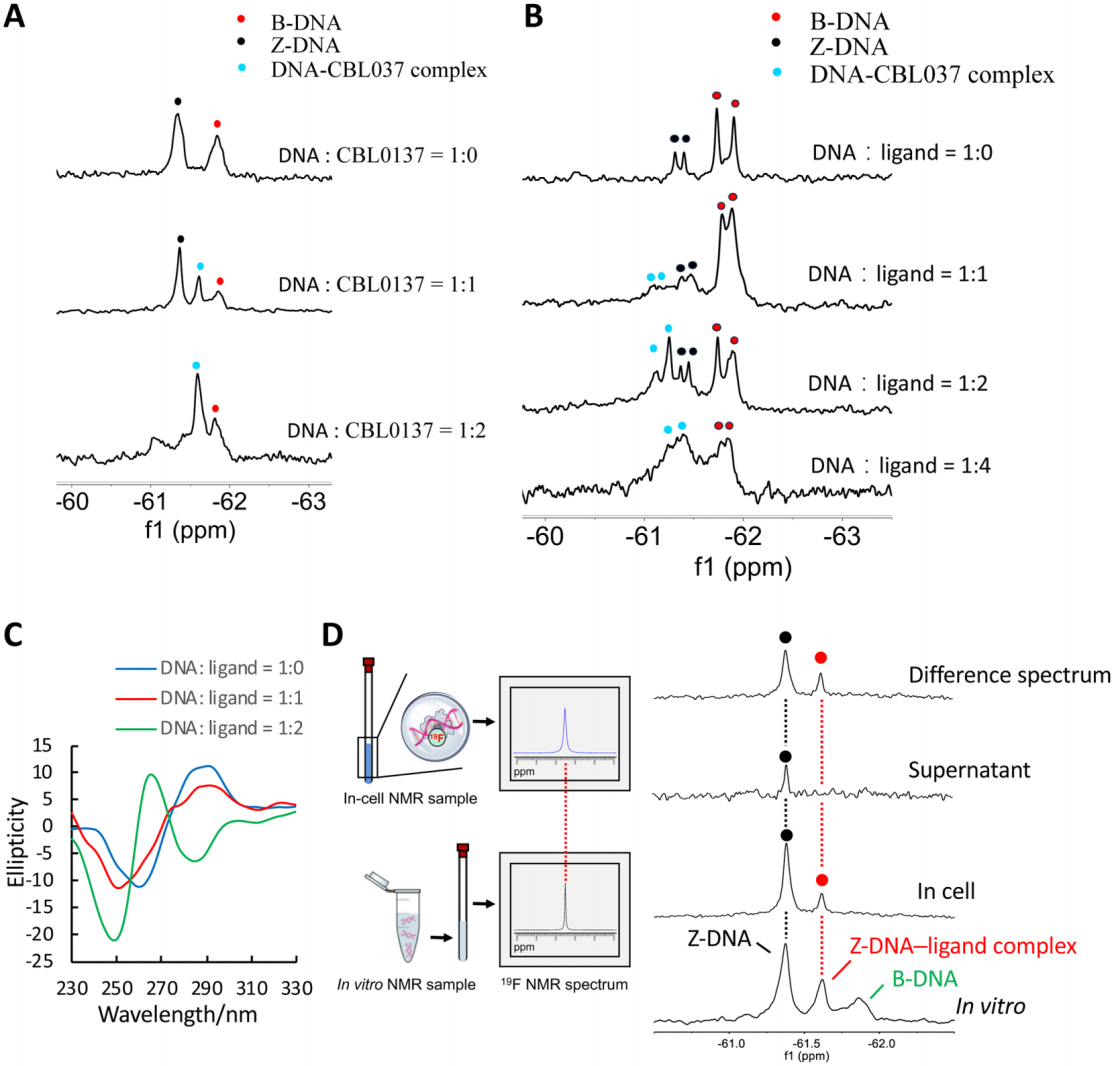
Characterization of CBL0137 ligand binding to Z-form DNA. **(A)**  ^19^F NMR spectra of 6-mer d(CGC^8F^GCG)_2_ titrated with CBL0137 in 10 mM NaCl, 5 mM Na-PO_4_ buffer (pH 7.0). The CBL0137-to-DNA molar ratio is indicated at the top. Red, black, and sky-blue signals correspond to B-DNA, Z-DNA, and the DNA–ligand complex, respectively. **(B)**  ^19^F NMR spectra of 8-mer d(C^8F^GCAC^8F^GCG)/d(CGCGTGCG) titrated with CBL0137 in 100 mM NaCl, 5 mM Na-PO_4_ buffer (pH 7.0). **(C)** CD spectra of 8-mer d(C^8F^GCAC^8F^GCG)/d(CGCGTGCG) with increasing concentrations of CBL0137 in 5 mM Na-PO_4_ buffer (pH 7.0). **(D)**  ^19^F NMR detection of the Z-DNA–CBL0137 complex in cells. Comparison of the in-cell spectrum with the *in vitro* reference enables reliable determination of intracellular structures. ^19^F NMR spectra showing *in vitro* B-DNA, *in vitro* Z-DNA, *in vitro* Z-DNA–CBL0137 complex, and their counterparts observed in living HeLa cells, in the supernatant, and in the difference spectrum (cell minus supernatant).

Thus, we could conclude that the ^19^F NMR signal is expected to be different for the free versus bound DNA, it allowed us to directly observe the DNA structural conversion from B- to Z-DNA by the ligand molecule. As shown in Fig. 2A, upon addition of CBL0137 to the 6-mer DNA sample, a new ^19^F NMR signal appeared at − 61.6 ppm, which was assigned to the DNA–ligand complex. Notably, the intensity of the Z-DNA signal decreased, indicating that CBL0137 binds to Z-DNA and forms a stable complex. In parallel, the intensity of the B-DNA signal also diminished, suggesting that the ligand promotes a B-to-Z conformational transition. Upon addition of two equivalents of CBL0137, the original Z-DNA signal completely disappeared, leaving only the DNA–ligand complex peak, whose intensity increased gradually, accompanied by a minor residual B-DNA signal. Furthermore, the 8-mer duplex exhibited a similar result (Fig. 2B), in which the DNA–ligand complex peaks were clearly observed, accompanied by a concomitant decrease in both the Z-DNA and B-DNA signals. Consistently, CD spectroscopy revealed that addition of equimolar CBL0137 (1:1) to the 8-mer duplex induced a characteristic spectral change near 285 nm (Fig. 2C). This effect became more pronounced at a higher CBL0137-to-DNA ratio (2:1), where a distinct negative band appeared around 285 nm, indicating that CBL0137 facilitates the B-to-Z transition. This is consistent with a higher binding constant *K_a_* = 1.35 × 10^5^ M^−1^ of CBL0137 and left-handed Z-form d(CGC^8F^GCG)_2_ complex compared to that of *K_a_* = 4.30 × 10^4^ M^−1^ of CBL0137 with right-handed B-form d(CGCGCG)_2_ complex (Supplementary Fig. S3).

In addition, we prepared a non–CG-repeat DNA sequence, d(CTT^8F^GCAAG)_2_, which is not expected to undergo a B–Z transition upon ligand binding. CD analysis showed a prominent B-form conformation, characterized by a strong positive band around 275 nm in the absence of ligand. Upon addition of CBL0137 at DNA/ligand ratios of 1:2 or 1:4, only a very weak signal near 295 nm was observed, indicating that d(CTT^8F^GCAAG)_2_ preferentially remains in the B-DNA conformation and is largely resistant to ligand binding (Supplementary Fig. S4A). ^19^F NMR spectroscopy revealed a single ^19^F resonance at − 61.91 ppm in the absence of ligand. After the addition of CBL0137, a new peak at − 60.94 ppm with much lower intensity appeared, which can be assigned to weak ligand-induced Z-DNA formation, consistent with the faint negative CD band observed at 295 nm (Supplementary Fig. S4B). Together, these results indicate that the B-form d(CTT^8F^GCAAG)_2_ duplex is intrinsically stable and largely resistant to ligand binding. Overall, these findings further demonstrate that CBL0137 exhibits a strong preference for binding and stabilizing Z-DNA over B-form DNA*in vitro*.

### Observation of the Z-DNA–CBL0137 complex in living cells by ^19^F NMR

Over the past decade, in-cell ^19^F NMR spectroscopy has been established as a powerful approach for probing nucleic acid and protein conformations under physiological conditions, owing to the high sensitivity and specificity of ^19^F in the absence of endogenous fluorine [19]. Because ^19^F chemical shifts are highly sensitive to local structural environments, distinct nucleic acid conformations can be reliably identified in cells by comparison with *in vitro* reference spectra. This strategy has been successfully applied to characterize a variety of nucleic acid structures in cells, including DNA and RNA G-quadruplexes [18, 20, 21], DNA–RNA hybrid G-quadruplexes [22], Z-DNA [12], and G-quadruplex–binding ligands [23].

As illustrated in Fig. 2D, comparison of the in-cell ^19^F NMR spectrum with the *in vitro* reference enables reliable identification of the intracellular DNA–ligand complex. The d(CGC^8F^GCG)_2_ duplex and ligand CBL0137 were introduced into HeLa cells using an SLO-based delivery method [19]. In-cell ^19^F NMR, two ^19^F signals were observed, with chemical shifts identical to those of Z-DNA and the Z-DNA–CBL0137 complex observed *in vitro* (Fig. 2D). After the measurement, the suspension was collected and analyzed by ^19^F NMR, in which almost no complex signal was detected in the supernatant, confirming that the observed signals originated from intracellular ^19^F-labeled DNA. To further exclude extracellular contributions, a difference spectrum between HeLa cells and the suspension was generated, eliminating supernatant-derived signals. These results provide the first direct evidence that CBL0137 binds to Z-DNA in living cells.

To further verify whether CBL0137 specifically binds Z-DNA rather than B-DNA in cells, we introduced the B-form DNA sequence d(CTT^8F^GCAAG)_2_ as a control into HeLa cells. In-cell ^19^F NMR spectra showed a single resonance whose chemical shift is consistent with that of B-DNA observed *in vitro* (Supplementary Fig. S4C). Analysis of the corresponding supernatant revealed only a much weaker signal, indicating that the majority of the detected ^19^F NMR signal originated from the ^19^F-labeled DNA inside the cells (Supplementary Fig. S4C), consistent with the difference spectrum between the cell and the suspension. These results indicate that CBL0137 does not appreciably associate with B-form DNA in cells. In contrast, clear signals corresponding to Z-DNA–CBL0137 complexes were observed for CG-repeat sequences (Fig. 2), demonstrating that CBL0137 preferentially binds Z-DNA over B-form DNA in the cellular environment. These in-cell observations are in strong agreement with the *in vitro* results (Fig. 2 and Supplementary Fig. S4).

### NMR spectroscopy reveals Z-form DNA bound to CBL0137

To elucidate the structure of the Z-DNA–CBL0137 complex, we first determined the solution structure of Z-form d(CGC^8F^GCG)₂ by NMR spectroscopy. Non-exchangeable proton resonances were assigned using 2D NOESY spectra in D₂O (Supplementary Fig. S5). Sequential NOE connectivities along the duplex, including C1(H6/H2′′)–G2(H8/H1′)–C3(H6/H2′′) and C5(H6/H2′′)–G6(H8/H1′), together with strong intranucleotide NOEs for G2 and G6, confirmed the *syn* glycosidic conformation of guanine residues and were consistent with a Z-DNA structure (Supplementary Fig. S5A and B; Supplementary Table S1). Characteristic upfield shifts of cytidine H2′ (1.70–1.80 ppm) and H5 (5.28–5.78 ppm) resonances further supported the Z-DNA conformation (Supplementary Fig. S5). Based on NOE-derived restraints (Supplementary Table S2), a refined structural ensemble was generated (Supplementary Fig. S5C), and the lowest-energy conformer clearly exhibited the hallmark zig-zag phosphate backbone of Z-DNA (Supplementary Figs. S5D and E and Supplementary Table S3). These results demonstrate that d(CGC^8F^GCG)₂ adopts a stable Z-DNA conformation under the experimental conditions.

**Figure 3.**
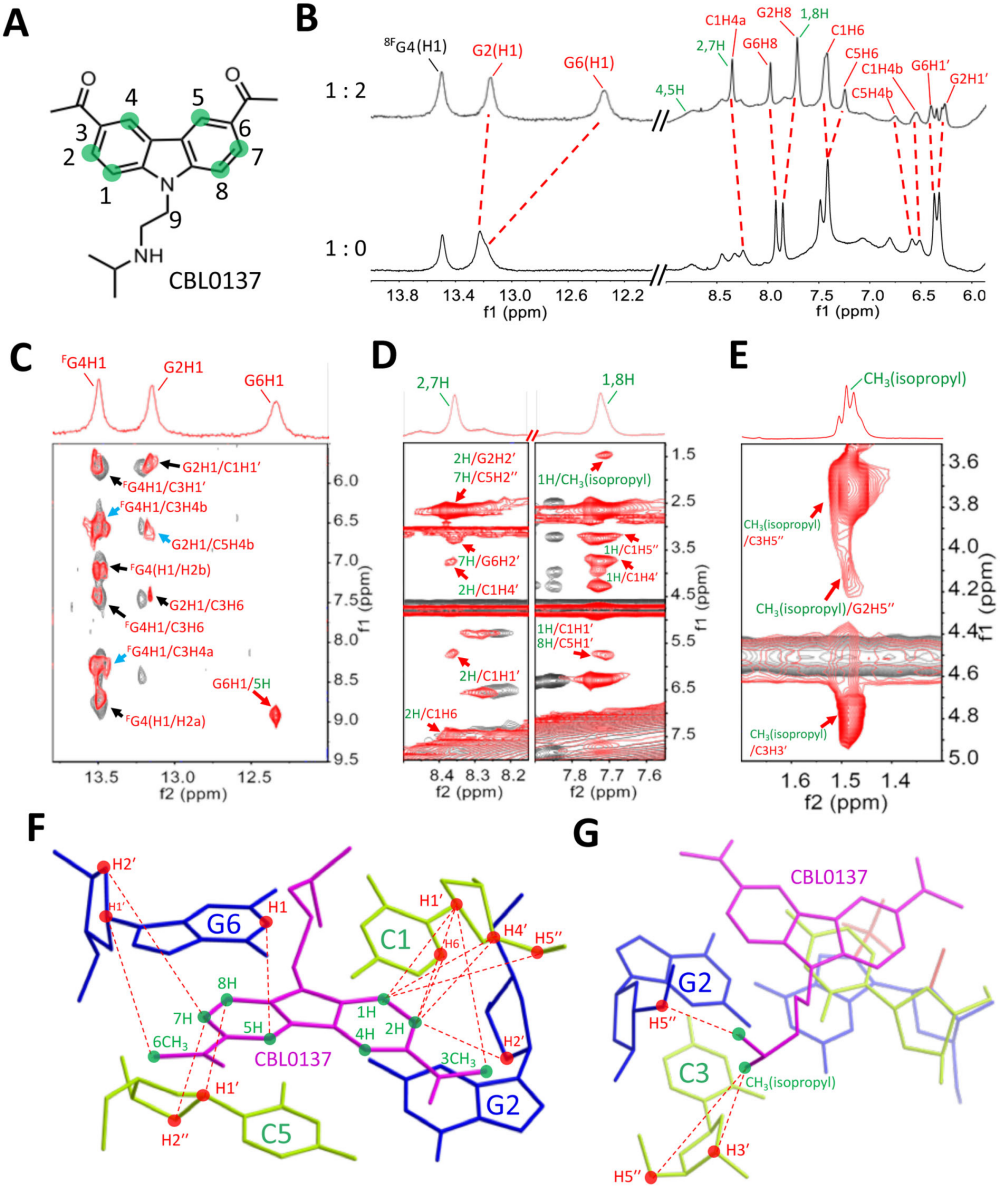
NMR spectroscopy study of CBL0137 binding to Z-form DNA. **(A)** Structure of CBL0137, containing a carbazole moiety with an N-side chain. Hydrogen atoms on the carbazole ring are labeled with green circles according to their positions. **(B)** 1D NMR spectra of Z-form DNA d(C1G2C3^8F^G4C5G6)_2_ with CBL0137 at 20°C in H_2_O/D_2_O (9:1). In the imino proton region, G2H1 and G6H1 exhibit upfield shifts upon complex formation (Z-DNA:ligand = 1:2), indicated by red dashed lines. In the amino and aromatic regions (including H1′), new peaks corresponding to the complex appear after titration, also marked by red dashed lines. Protons from CBL0137 are shown in green. (**C**–**E**) Overlay of NOESY spectra. Red signals correspond to the Z-DNA–CBL0137 complex, whereas black signals represent Z-DNA alone as a control. **(C)** Imino proton region, showing Watson–Crick base pairing supported by NOEs of ^8F^G4H1/C3H4b, G2H1/C5H4b, and ^8F^G4H1/C3H4a (blue arrows). A cross peak between G6H1 and 5H indicates interaction with CBL0137 (red arrow). **(D)** Series of NOEs involving 1,8H and 2,7H of CBL0137, demonstrating ligand–DNA interactions (red arrows). **(E)** The CH_3_ (isopropyl) group of CBL0137 exhibits NOEs to DNA protons (red arrows). **(F, G)** Intermolecular NOE contacts position CBL0137 between C1:G6 and G2:C5 base pairs, viewed from the major **(F)** and minor **(G)** grooves. NOE-derived interactions between DNA and CBL0137 are shown as red dashed lines. In all panels, red labels represent DNA protons and green labels represent ligand protons.

The well-defined Z-form DNA of d(CGC^8F^GCG)₂ allows us to investigate the Z-DNA–CBL0137 complex. Initial 1D proton NMR experiments were performed, in which titration of CBL0137 into DNA at a 2:1 ligand-to-duplex ratio led to the emergence of a new set of peaks corresponding to the complex (Fig. 3A and B), indicating that CBL0137 binding occurs in a slow exchange regime on the NMR timescale. Complete ^1^H chemical shift assignments for both CBL0137 and DNA in the complex are listed in Supplementary Tables S5–S7. Notably, a pronounced upfield shift of G6H1 (Δδ = –0.84 ppm) was observed, representing a typical ligand interaction. In addition, G2H1 showed an upfield shift (Δδ = –0.06 ppm), while ^8F^G4H1 exhibited no change, collectively suggesting that the binding site of CBL0137 lies near G2 and G6 rather than ^8F^G4 (Fig. 3B). Furthermore, a series of Z-DNA proton resonances displayed altered chemical shifts upon ligand titration. These signals were assigned to residues C1, G2, C5, and G6, verifying that CBL0137 localizes to the Z-DNA region encompassing the C1:G6 and G2:C5 base pairs (Fig. 3B).

Moreover, overlay of the 2D NOESY spectra provided additional structural insights into the CBL0137–DNA complex (Fig. 3C–E). NOEs involving ^8F^G4H1 were highly consistent with Z-DNA alone, indicating that ligand binding does not alter the overall topology at the ^8F^G4 residue. Cross peaks of G2H1 exhibited a slight upfield shift upon ligand binding, while a distinct NOE between G6H1 and the ligand’s 5H verified direct interaction of the carbazole moiety with the G6 base (Fig. 3C). Numerous intermolecular NOEs were identified (Fig. 3D). For instance, ligand 2H showed NOEs to C1H6, C1H1′, and G2H2′, and a characteristic cross peak between 3CH_3_ and C1H1′ was also observed (Fig. 3D and Supplementary Fig. S6), confirming that the ligand intercalates between C1 and G2. Additionally, NOEs of ligand 7H with C5H2′′ and G6H2′, together with 6CH_3_/G6H1′, demonstrate that the carbazole moiety inserts into the cavity between C1:G6 and G2:C5 base pairs (Fig. 3D, Supplementary Fig. S6). Further NOEs between ligand 1H and 8H with C1H1′, C1H4′, C1H5′′, and C5H1′ suggest proximity to the sugar backbone toward the minor groove. Finally, the ligand’s N-side chain isopropyl group showed NOEs with G2H5′′, C3H3′, and C3H5′′, indicating minor groove accommodation (Fig. 3E). A comprehensive interaction map of intermolecular NOE contacts (Fig. 3F and G; Supplementary Table S8) reveals that the carbazole moiety interacts between C1:G6 and G2:C5, while the N-side chain of the isopropyl group contacts G2 and C3.

Furthermore, sequential assignments from C1 to C3 and from C5 to G6 in the Z-form allowed completion of the pathway: C1(H6/H5′′) → G2(H8/H2′′) → C3(H6/H5′′) and C5(H6/H5′′) → G6(H8/H2′′) (Supplementary Fig. S7), indicating a sequence-specific connectivity characteristic of left-handed Z-helices. Several distinctive NOEs of Z-DNA were observed, such as intranucleotide G2(H8/H1′) and G6(H8/H1′) cross-peaks, which reflect the *syn* conformation of the dG residues. The H5 protons of C3 and C5 showed upfield shifts (5.27 and 5.21 ppm, respectively). Cross-peaks were also observed between C1H5 and the C5 amino proton (C1H5/C5H4b) and between C3H5 and the C3 amino proton (C3H5/C3H4b), consistent with inter-strand stacking at CpG steps of a standard feature of Z-DNA [12, 24]. Collectively, these observations confirm that the DNA duplex retains its Z-form structure upon binding with the ligand.

### Solution structure of Z-form DNA bound to CBL0137

**Figure 4.**
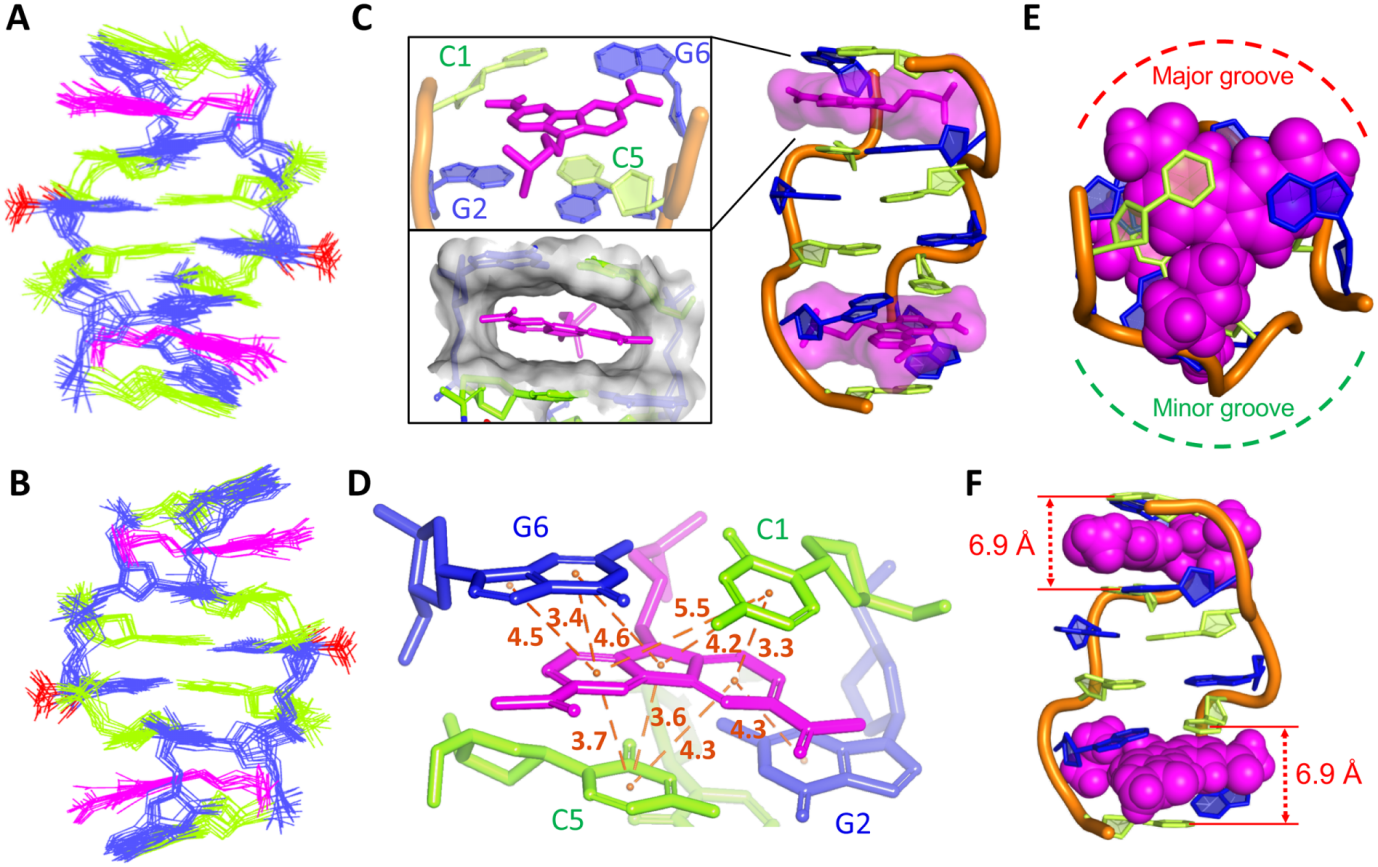
Molecular model of CBL0137 binding to Z-form DNA. **(A, B)** Ensemble of 10 conformers (line representation) viewed from the major **(A)** and minor **(B)** grooves. **(C)** Solution NMR structure of the CBL0137–DNA complex (cartoon representation). CBL0137 is shown in pink sticks with a transparent molecular surface, bound symmetrically to Z-DNA (green, dC residues; blue, dG residues; orange ribbon, phosphate backbone). Upper inset: ligand intercalation between C1:G6 and G2:C5 base pairs, forming a sandwich structure. Lower inset: solvent-excluded hydrophobic cavity (gray) of the DNA pocket at C1:G6 and G2:C5, where CBL0137 localizes into the cleft pocket. **(D)** Expanded view showing multiple π–π stacking interactions (orange dashed lines) between the carbazole moiety and base pairs C1:G6 and G2:C5, with interatomic distances indicated. **(E)** Carbazole moiety (pink CPK) protrudes into the major groove (red dashed line), while the N-side chain extends into the minor groove (green dashed line). **(F)** Insertion of CBL0137 increases the helical rise between C1:G6 and G2:C5 base pairs to 6.9 Å.

The NMR solution model of the Z-DNA–CBL0137 complex was constructed using NOE-derived restraints (Supplementary Table S2). The lowest-energy refined structures of the complex are presented in Fig. 4A and B. Two ligands symmetrically intercalate into the cleft formed by C1:G6 and G2:C5 base pairs at the CpG step (Fig. 4C). Intercalation of the aromatic carbazole ring creates a hydrophobic cavity stabilized by extensive π–π stacking with adjacent base pairs (Fig. 4C and D), consistent with the lower RMSD of the complex compared to Z-DNA alone (Supplementary Table S2). A top view shows the carbazole moiety protruding toward the major groove, while the N-side chain extends into the minor groove (Fig. 4E). Ligand insertion also induces a significant increase in helical rise between C1:G6 and G2:C5 (6.9 versus 3.9 Å in free Z-DNA; Fig. 4F, Supplementary Tables S4 and S10). Despite ligand intercalation, inter-stranded base pair stacking is maintained at CpG but not GpC steps, consistent with canonical Z-DNA (Supplementary Fig. S8). Collectively, these results demonstrate that CBL0137 binds Z-DNA without disrupting its characteristic zig-zag backbone, as further supported by altered helical parameters indicating base pairs shift closer to the Z-axis upon ligand binding (Supplementary Fig. S9; Supplementary Tables S9 and  S10). These findings provide the first atomic-level insight into how CBL0137 recognizes and stabilizes Z-DNA, establishing a structural basis for its therapeutic potential.

### Zig-zag pattern of the phosphate backbone localizes CBL0137 in Z-DNA

**Figure 5.**
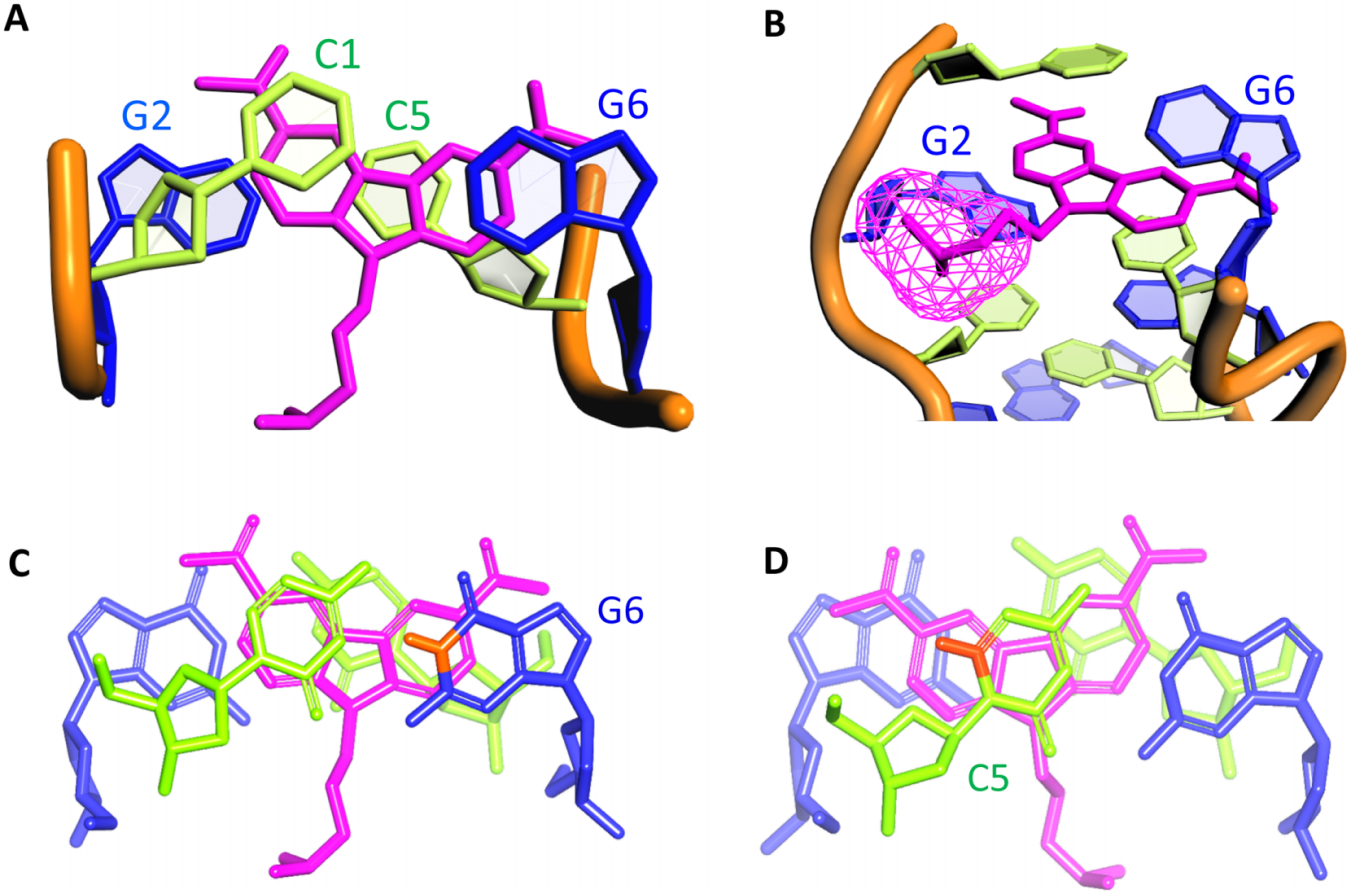
Localization of CBL0137 within Z-form DNA. **(A)** The carbazole moiety preferentially inserts into the cleft pocket comprising bases C1, C5, and G6, while remaining distant from G2. **(B)** The sugar–phosphate backbone at G2 adopts a more open conformation, providing space to accommodate the N-side chain of the ligand (surface highlighted with mesh representation) and guiding its embedding orientation. **(C, D)** The NH1 of G6 (orange label, **C**) and H6 of C5 (orange label, **D**) are positioned above the aromatic ring of the carbazole moiety, experiencing shielding by the π-electron cloud during NMR measurements.

In the solution NMR structure of the Z-DNA–CBL0137 complex, the carbazole moiety of CBL0137 was found to embed into the pocket between the C1:G6 and G2:C5 base pairs, preferentially localizing near C1, C5, and G6 rather than G2 (Fig. 5A). This is consistent with the NOE results, which revealed more extensive contacts with C1, C5, and G6 than with G2 (Fig. 3F). The asymmetric localization of the ligand can be attributed to the characteristic zig-zag phosphate backbone of Z-DNA. Specifically, the phosphate group at the 5′-end of G2 provides a favorable site for the N-side chain of CBL0137, whereas the phosphate group of G6 does not (Fig. 5B). Consequently, the carbazole moiety orients toward the bases of C1, C5, and G6 while remaining distant from G2. This orientation places G6H1 directly above the aromatic ring of the carbazole (Fig. 5C), leading to a pronounced ring-current effect that reinforces shielding and explains the strong upfield shift of G6H1 (Fig. 3B). Similarly, C5H6 exhibited weaker NOEs to its sugar protons compared with C3H6, likely reflecting carbazole-induced shielding, as C5H6 is positioned within the aromatic ring current region (Fig. 5D, Supplementary Fig. S7).

**Figure 6.**
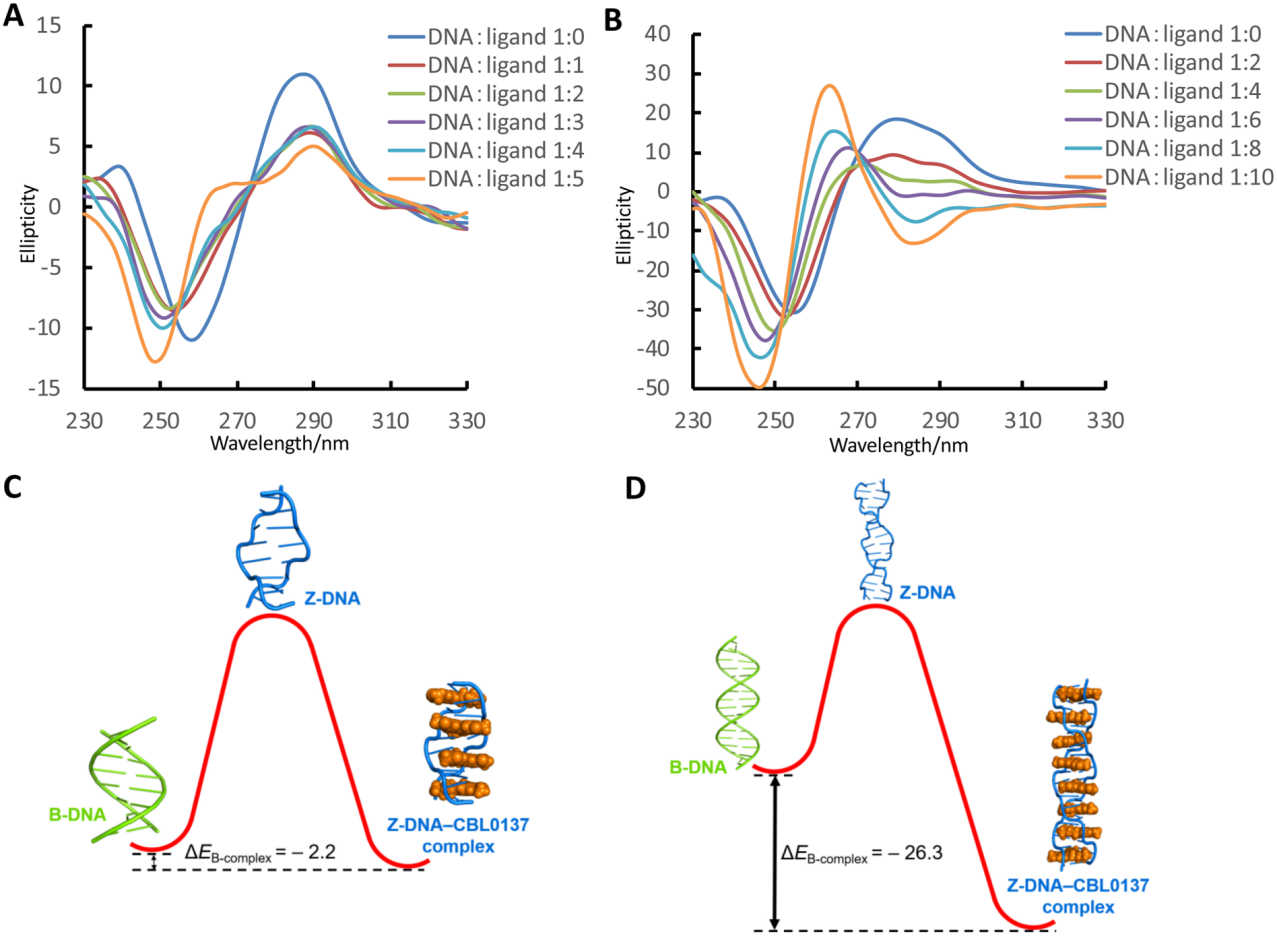
CBL0137 promotes the B–Z transition more efficiently in longer DNA duplexes. **(A)** CD spectra of the native 8-mer duplex d(CGCACGCG)/d(CGCGTGCG) and **(B)** 16-mer duplex d(CGCACGCGCGCACGCG)/d(CGCGTGCGCGCGTGCG) recorded with increasing concentrations of CBL0137 in 5 mM Na-PO_4_ buffer (pH 7.0). **(C)** For the 8-mer duplex, CBL0137 binding lowers the energy of the Z-DNA form to –2.2 kcal mol⁻¹, making it energetically more favorable than the B-form. **(D)** For the 16-mer duplex, CBL0137 binding markedly decreases the Z-DNA energy to –26.3 kcal mol⁻¹ relative to the B-form, thereby enhancing the overall stability of the duplex system.

Considering the long DNA present in the intracellular environment, we employed a longer duplex to examine the B–Z transition induced by CBL0137. In the CD experiment, we found that CBL0137 more readily converts the16-mer duplex without modified ^8F^G d(CGCACGCGCGCACGCG)/d(CGCGTGCGCGCGTGCG) into Z-DNA compared with the shorter 8-mer duplex d(CGCACGCG)/d(CGCGTGCG) (Fig. 6A and B). This suggests that longer DNA provides more potential binding sites for CBL0137, thereby facilitating the B–Z transition (Fig. 6B). These findings are consistent with the proposed role of CBL0137 in enhancing cancer immunotherapy through the induction of Z-DNA formation [10].

To elucidate the energetic basis of CBL0137 binding and Z-DNA stabilization, molecular energy calculations were performed. The 8-mer duplex required higher energy to adopt the Z-form than the B-form (+18.49 kcal/mol, Supplementary Table S12). Upon complex formation, the Z-DNA energy decreased markedly and became lower than that of B-DNA (–2.20 kcal/mol; Fig. 6C), indicating that CBL0137 binding drives the B–Z equilibrium toward a more stable Z-DNA–ligand complex. The 16-mer duplex similarly exhibited higher energy in the Z-form (+14.77 kcal/mol, Supplementary Table S12), but with a smaller B–Z energy gap than the 8-mer, suggesting that longer DNA is intrinsically more prone to adopt the Z-conformation. Upon CBL0137 binding, the Z-form energy dropped substantially (–26.30 kcal/mol; Fig. 6D), demonstrating that longer DNA provides additional binding sites for CBL0137, thereby promoting the B–Z transition and stabilizing the Z-DNA structure.

To characterize the binding behavior of CBL0137 to Z-DNA, we performed detailed biophysical analyses (Supplementary Fig. S10). CD-based Job plot analyses revealed a clear tipping point at the ratio of *n* = 4 for the 8-mer duplex DNA, indicating that four CBL0137 molecules bind to one 8-mer duplex (Supplementary Fig. S10E). Consistently, titration of the 16-mer DNA reached equivalence at *n* = 8, demonstrating that eight ligand molecules interact with the longer duplex (Supplementary Fig. S10F). Moreover, the 16-mer DNA exhibited a larger change in ellipticity (Δθ) at 285 nm compared with the 8-mer DNA (−31.18 versus − 19.08) upon reaching binding equivalence (Supplementary Fig. S10A and B). This result indicates a more pronounced B–Z transition for the longer DNA, consistent with the observations in Fig. 6A and B, and suggests that longer DNA can accommodate more ligands, thereby favoring stabilization of the Z-DNA conformation.

To quantitatively compare binding affinities, we determined the association constants (*K*_a_) (Supplementary Fig. S10C and D). A higher binding affinity was observed for the 16-mer DNA (*K*_a_ = 7.6 × 10^5^ M^−1^) compared with the 8-mer DNA (*K*_a_ = 5.5 × 10^5^ M^−1^). This difference can be attributed to the fact that the 16-mer consists of two repeats of the 8-mer sequence, allowing preservation of strong ligand–DNA interactions.

## Discussion

In this study, we constructed a stable Z-form DNA duplex, d(CGC^8F^GCG)₂, using 8-trifluoromethyl-2′-deoxy-2′-fluoroguanosine, which adopts the *syn* conformation with a C3′-endo sugar pucker to promote Z-helix formation [12, 15]. We then investigated its interaction with the curaxin ligand CBL0137 and comprehensively characterized the binding landscape through high-resolution NMR analysis. The carbazole moiety intercalates into the CpG step between the C1:G6 and G2:C5 base pairs via hydrophobic and π–π stacking interactions, while the N-side chain aligns within the minor groove. The zig-zag phosphate backbone of Z-DNA generates asymmetric spatial constraints, particularly at the 5′-end of G2, favoring localization of the side chain and driving the carbazole toward C1, C5, and G6 rather than G2. These insights reveal a unique recognition mechanism, offering guiding principles for designing next-generation Z-DNA ligands. For example, optimization of the flexibility and length of the N-side chains may facilitate better accommodation within the rigid minor groove of Z-DNA, which adopts a characteristic zig-zag geometry. In addition, increasing the positive charge density of the side chains could strengthen electrostatic interactions with the negatively charged phosphate backbone, thereby enhancing binding affinity. Furthermore, our structural analysis revealed that the G2 base is not fully engaged in ligand interactions, as the carbazole moiety primarily orients toward the C1, C5, and G6 residues, leaving G2 unutilized. Expanding the planar aromatic surface of the ligand may therefore promote additional π–π stacking interactions with base pairs, leading to improved complex stability.

Compared with canonical B-DNA, Z-DNA exhibits a larger helical rise and more pronounced slide and roll parameters, particularly at CpG steps (Supplementary Fig. S11). These structural features lead to weakened inter–base pair π–π stacking interactions, as evidenced by increased inter-base distances and reduced hydrophobic interactions (Supplementary Fig. S12). As DNA duplex stability strongly depends on base-stacking interactions, these characteristics indicate that Z-DNA is intrinsically higher in energy than B-form DNA (Fig. 6C and D). Upon binding, CBL0137 intercalates into the inner hydrophobic pocket between base pairs of Z-DNA, enhancing intermolecular interactions and shortening inter-base distances. This intercalation effectively reinforces base stacking and lowers the overall energy of the complex, thereby stabilizing the Z-DNA structure (Figs 4D, 6C, and D). Moreover, the wider minor groove of Z-DNA provides additional space to accommodate side chains (Supplementary Table S11). The binding of CBL0137 to and stabilization of Z-DNA highlight enormous potential for biomedical applications. Our findings provide direct evidence for the underlying mechanism by which CBL0137 preferentially stabilizes Z-DNA over B-DNA, consistent with its ability to promote the B–Z transition more efficiently in longer DNA duplexes [10]. Finally, in-cell ^19^F NMR experiments confirmed the presence of Z-DNA in living cells and, importantly, revealed a distinct signal corresponding to the Z-DNA–CBL0137 complex. To our knowledge, this represents the first direct observation of a Z-DNA–ligand complex inside cells.

Understanding the properties of ligand binding to Z-DNA is important for elucidating the molecular mechanism underlying CBL0137-induced B–Z transition in cells. Our results demonstrate that longer DNA sequences are more prone to undergo B–Z conversion by accommodating a greater number of ligand molecules (Supplementary Fig. S10). Furthermore, Z-DNA exhibits a higher binding affinity for CBL0137 than B-DNA (Supplementary Fig. S3). Considering that genomic DNA is extremely long (∼6 × 10^9^ base pairs) and contains numerous CG-alternating sequences that favor B–Z transition, these findings suggest that abundant binding sites are available for ligand interaction and Z-DNA stabilization. This provides a molecular-level rationale for CBL0137-induced stabilization of Z-DNA and subsequent activation of the necroptosis pathway in cancer immunotherapy [10].

Recent studies have shown that Z-RNA generated during viral infection, such as influenza virus infection, activates RIPK3 and MLKL, leading to nuclear envelope rupture and subsequent inflammatory responses, including neutrophil recruitment [9, 25]. Together with previous reports on Z-DNA–induced necroptosis [10], these findings collectively demonstrate that Z-form nucleic acid duplexes–either DNA or RNA–possess distinctive structural features capable of eliciting strong physiological responses via ZBP1 signaling.

In summary, we present the first atomic-resolution model of a Z-DNA–CBL0137 complex, providing a detailed molecular basis for ligand recognition and stabilization [26–31]. These findings open new avenues for the rational design of small molecules targeting Z-DNA, with promising implications for biological and biomedical applications.

## Supplementary Material

gkag104_Supplemental_File

## Data Availability

All data that support the findings of this study are available in the Supporting Information of this article. The NMR data of the Z–DNA–ligand complex were deposited in the Biological Magnetic Resonance Bank under BMRB ID 36774. NMR coordinate of Z-DNA–ligand complex was deposited in PDB bank (code: 9VZW).
