# Supplementary material for: Solution structure of Z-form DNA bound to a curaxin ligand CBL0137
Source: Nucleic Acids Res. 2026 Feb 10;54(4):gkag104. doi: 10.1093/nar/gkag104 (PMC12887531; doi:10.1093/nar/gkag104)
Supplement: gkag104_Supplemental_File [file gkag104_supplemental_file.pdf]

Table of contents

Synthesis of compounds 1–6 **2**

Supplementary Figures and Tables **5**

References **21**

Synthesis of compounds 1–6

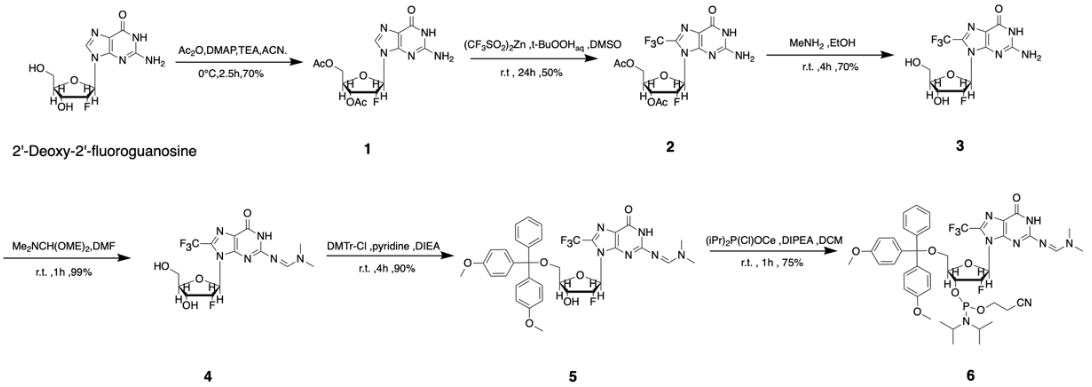

Scheme S1. Synthesis of ^{8F}G and relative phosphoramidite compound.

3',5'-O-acetyl-2'-Deoxy-2'-fluoroguanosine (1)

2'-Deoxy-2'-fluoroguanosine (2000 mg, 7.0 mmol), trimethylamine (7.87 mL, 55.7 mmol) and 4-dimethylaminopyridine (93 mg, 0.75 mmol) were dissolved in 25 mL anhydrous acetonitrile, acetic anhydride (2.22 mL, 22.2 mmol) was added dropwise and the mixture reacted for 1.5 hours at 0 °C and another 1 hour at room temperature. The reaction was quenched by additional methanol (2.32 mL, 57.45 mmol). The volume was reduced to 1/3 using a rotary evaporator and diethyl ether was added dropwise to induce precipitation of a fine white powder. The product was collected by filtration, washed with diethyl ether, and then stirred for 2 hours in acetone (30 mL) at 50 °C. The filtrate produced 1800 mg (70% yield) of a fine white powder.

^{1}H NMR (500 MHz, DMSO) δ 10.73 (s, 1H), 7.88 (s, 1H), 6.52 (s, 2H), 6.11 (dd, J = 18.5, 3.3 Hz, 1H), 5.72 (ddd, J = 51.5, 5.2, 3.3 Hz, 1H), 5.54 (dt, J = 14.6, 5.7 Hz, 1H), 4.36 (td, J = 8.1, 3.1 Hz, 2H), 4.26 – 4.19 (m, 1H), 2.14 (s, 3H), 2.03 (s, 3H). HRMS (ESI) for C14H16FN5O6 $[M+H]^+$: Calcd. 370.1085; Found 370.1157.

3',5'-O-acetyl-8-trifluoro-2'-Deoxy-2'-fluoroguanosine (2)

2'-Deoxy-2'-fluoroguanosine (1800 mg, 4.9 mmol) and zinc trifluoromethanesulfinate (5800 mg, 15.7 mmol) were dissolved in dimethyl sulfoxide (20 ml) and vigorous stirring. When the clear solution was formed, the tert-butyl hydroperoxide (70% solution in water, 3.30 mL, 24.7 mmol) was divided into 10 portions (330 µL each) and added sequentially at 20-minute intervals. The reaction mixture gradually turn yellow during addition of tert-Butyl hydroperoxide and continue reacted for 24 hours at room temperature. The reaction mixture was poured into 300 mL of water and extracted with dichloromethane (3 × 80 mL). The combined organic layers were washed sequentially with water (approximately 3 × 50 mL) and saturated brine (approximately 50 mL), then dried over anhydrous sodium sulfate. The drying agent was filtered off and washed with dichloromethane. The filtrate was concentrated under reduced pressure. The resulting oily residue was purified by medium pressure liquid chromatography (MPLC) using 5% methanol in dichloromethane (v/v) as the eluent to afford product 2 (1070 mg, 50% yield).

^{1}H NMR (500 MHz, DMSO) δ 11.13 (s, 1H), 6.83 (s, 2H), 6.19 – 5.80 (m, 2H), 5.69 (ddd, J = 17.5, 8.0, 5.6 Hz, 1H), 4.58 – 4.34 (m, 2H), 4.19 (dd, J = 12.8, 7.3 Hz, 1H),2.15 (s, 3H), 1.98 (s, 3H). HRMS (ESI) for $C_{15}H_{15}F_4N_5O_6$ $[M+H]^+$: Calcd. 438.0958; Found 438.1033.

8-trifluoromethyl-2'-deoxy-2'-fluoroguanosine (3)

3',5'-O-acetyl-8-trifluoro-2'-Deoxy-2'-fluoroguanosine (1070 mg, 2.5 mmol) was placed in 200 mL round bottom flask. Methylamine (33% in ethanol, 12.96 mL, 124.3 mmol) was added for getting reaction mixture, and the mixture reacted for 4 hours at room temperature. The reaction solution was concentrated in vaco and the residue was purified by MPLC with the mixture of methanol in dichloromethane (10%, v/v). The product 3 was giving as yellow solid (600 mg, 70%).

^{1}H NMR (500 MHz, DMSO) δ 10.89 (s, 1H), 6.83 (s, 2H), 5.88 (dd, *J* = 20.9, 2.9 Hz, 1H), 5.71 (ddd, *J* = 53.7, 5.4, 2.9 Hz, 1H), 5.64 – 5.50 (m, 1H), 4.91 (s, 1H), 4.60 (dt, *J* = 16.6, 6.1 Hz, 1H), 3.93 (td, *J* = 6.7, 3.2 Hz, 1H), 3.71 (dd, *J* = 12.2, 3.3 Hz, 1H), 3.56 (dd, *J* = 12.2, 6.4 Hz, 1H).HRMS (ESI) for $C_{11}H_{11}F_4N_5O_4$ [M-H]$^-$: Calcd. 352.0747; Found 352.0622.

N^2-dimethylformamidyl-8-trifluoromethyl-2'-Deoxy-2'-fluoroguanosine (4)

2'-Deoxy-2'-fluoro8-trifluoromethylguanosine (600 mg, 1.7 mmol) and N,N-dimethylformamide dimethyl acetal (1.62 mL, 12.0 mmol) was dissolved in anhydrous dimethylformamide (10 mL). The mixture was reacted for 1 hour at room temperature and the solvent was evaporated *in vacuo* for getting residue. The residue was purified by MPLC with mixture of methanol in dichloromethane (10%, v/v), the product 4 was giving as white solid (700 mg, 99%).

^{1}H NMR (500 MHz, DMSO) δ 11.77 (s, 1H), 8.59 (s, 1H), 7.96 (s, 1H), 5.96 – 5.88 (m, 1H), 5.77 (dd, J = 5.3, 3.0 Hz, 1H), 5.71 (d, J = 6.2 Hz, 1H), 3.93 (dt, J = 7.1, 3.5 Hz, 1H), 3.73 (ddd, J = 12.4, 5.1, 2.9 Hz, 1H), 3.56 (ddd, J = 12.0, 6.8, 4.7 Hz, 1H), 3.19 (s, 3H), 3.08 (s, 3H), 2.90 (s, 4H), 2.74 (s, 3H). HRMS (ESI) for $C_{14}H_{16}F_4N_6O_4$ [M-H]$^-$: Calcd. 407.1169; Found 407.0992.

N^2-dimethylformamidyl-8-trifluoromethyl-5'-*O*-(4,4'-dimethoxytrityl)-2'-deoxy-2'-fluoroguanosine (5)

Compound 4 (700 mg, 1.7 mmol) and 4,4'-dimethoxytrityl chloride (1148.8 mg, 3.4 mmol) were dissolved in 10 mL anhydrous pyridine and the mixture stirred for 4 hours at room temperature. The solvent was concentrated *in vacuo* and the residue was purified by MPLC with mixture of methanol in dichloromethane (3%, v/v). The product was giving as white foam (1100 mg, 90%).

^{1}H NMR (500 MHz, DMSO) δ 11.87 (s, 1H), 8.58 (dt, J = 4.2, 1.8 Hz, 2H), 8.45 (s, 1H), 7.83 – 7.75 (m, 1H), 7.39 (ddd, J = 7.6, 4.3, 1.5 Hz, 2H), 7.23 – 7.13 (m, 5H), 7.13 – 7.03 (m, 4H), 6.80 – 6.73 (m, 4H), 6.20 – 5.85 (m, 2H), 5.74 (d, J = 6.5 Hz, 1H), 5.13 – 4.95 (m, 1H), 4.35 (t, J = 5.1 Hz, 0H), 4.12 – 3.96 (m, 1H), 3.72 (s, 3H), 3.72 (s, 4H), 3.07 (s, 3H), 3.06 (s, 3H). HRMS (ESI) for $C_{35}H_{34}F_4N_6O_6$ [M-H]$^-$: Calcd. 709.2476; Found 709.2145.

3'-*O*-phosphoramidite-N^2-dimethylformamidyl-8-trifluoromethyl-5'-*O*-(4,4'-dimethoxytrityl)-2'-deoxy-2'-fluoroguanosine (6)

Compound 5 (1100 mg, 1.5 mmol) co-evaporated with 5 mL anhydrous acetonitrile by three times and dissolved in 8 mL anhydrous dichloromethane. Diisopropylethylamine (0.98 mL, 5.6 mmol) was added. After 5 minutes, 2-cyanoethyl-N,N-diisopropylamidochlorophosphoramidite (0.62 mL, 3.0 mmol) also was added, the mixture was reacted for 1.5 hours at room temperature. The reaction mixture was extracted by

dichloromethane and organic layer concentrated *in vacuo*, the residue was purified by MPLC with mixture of ethyl acetate in hexane (100%, v/v). The product was giving as white foam (1100 mg, 70%).

^{1}H NMR (500 MHz, DMSO) δ 11.91 (s, 1H), 8.43 (d, J = 30.6 Hz, 1H), 7.20 – 7.06 (m, 7H), 6.85 – 6.71 (m, 4H), 6.14 – 6.02 (m, 2H), 5.38 – 5.12 (m, 1H), 4.22 – 4.14 (m, 1H), 4.05 (dddt, J = 37.5, 10.5, 7.7, 5.9 Hz, 1H), 3.72 (dd, J = 4.4, 1.4 Hz, 7H), 3.62 – 3.35 (m, 3H), 3.11 (s, 1H), 2.89 (t, J = 5.9 Hz, 1H), 2.83 (d, J = 5.4 Hz, 4H), 2.73 (s, 0H), 2.60 (t, J = 6.1 Hz, 1H), 2.18 (s, 4H), 1.27 – 1.15 (m, 17H), 1.11 – 1.04 (m, 10H), 0.89 (d, J = 6.8 Hz, 3H).HRMS (ESI) for $C_{44}H_{51}F_{4}N_{8}O_{7}P$ $[M-H]^{-}$: Calcd. 909.3554; Found 909.3356.

Supplementary Figures and Tables

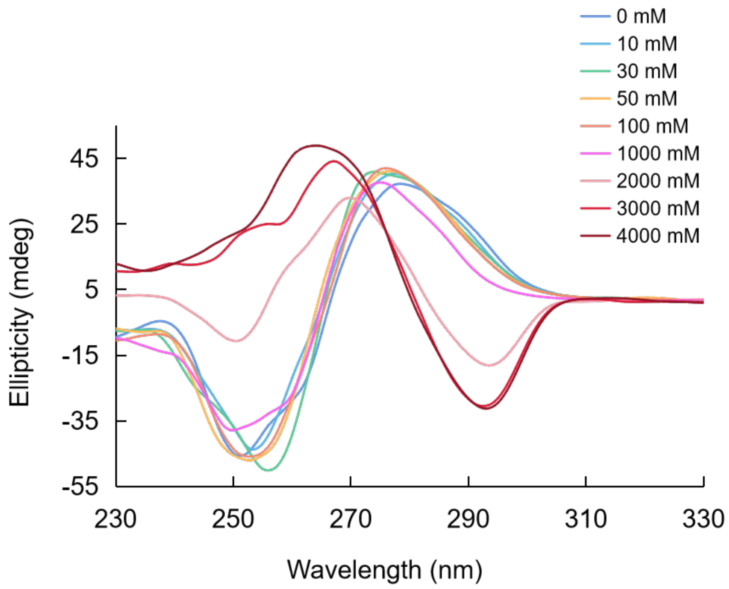

Figure S1. CD spectra of ^{2F}**G** modified DNA d(C^{2F}**G**CAC^{2F}**G**CG)/d(CGCGTGCG) in 1 mM Na-PO_4 buffer (pH 7.0), at 283 K. Various NaCl concentrations are indicated. A negative Cotton effect appears at 295 nm indicating a Z-DNA.

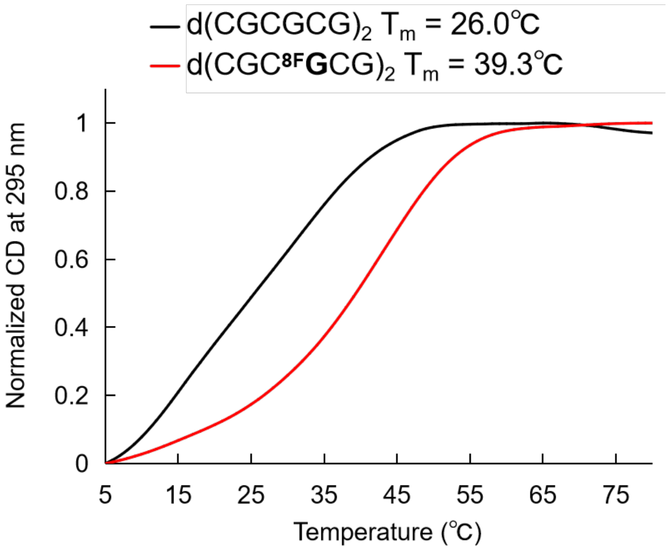

Figure S2. Melting profiles of the CD signals versus temperature, using native $d(CGCGCG)_2$ and modified d(CGC^{8F}**G**CG)$_2$. The profiles were obtained by plotting the normalized CD signals in CD spectra as a function of temperature.

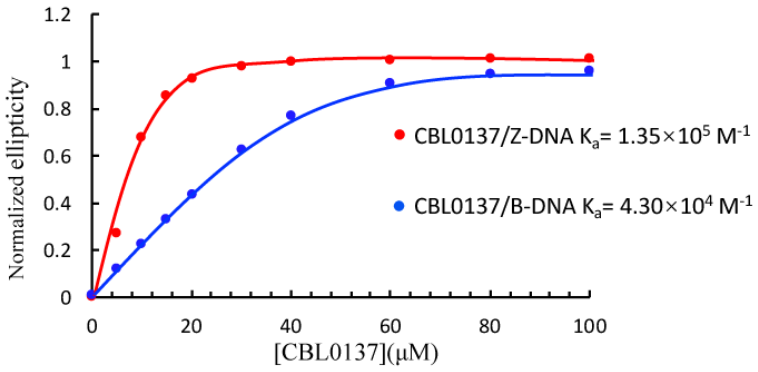

Figure S3. Titration data of CBL0137 and DNA derived by monitoring the wavelength at 280 nm of CD. The solid lines are the theoretical fit of the data. The binding constants of CBL0137 and Z-form d(CGC^{8F}**G**CG)$_2$, CBL0137 and B-form d(CGCGCG)$_2$ are indicated.

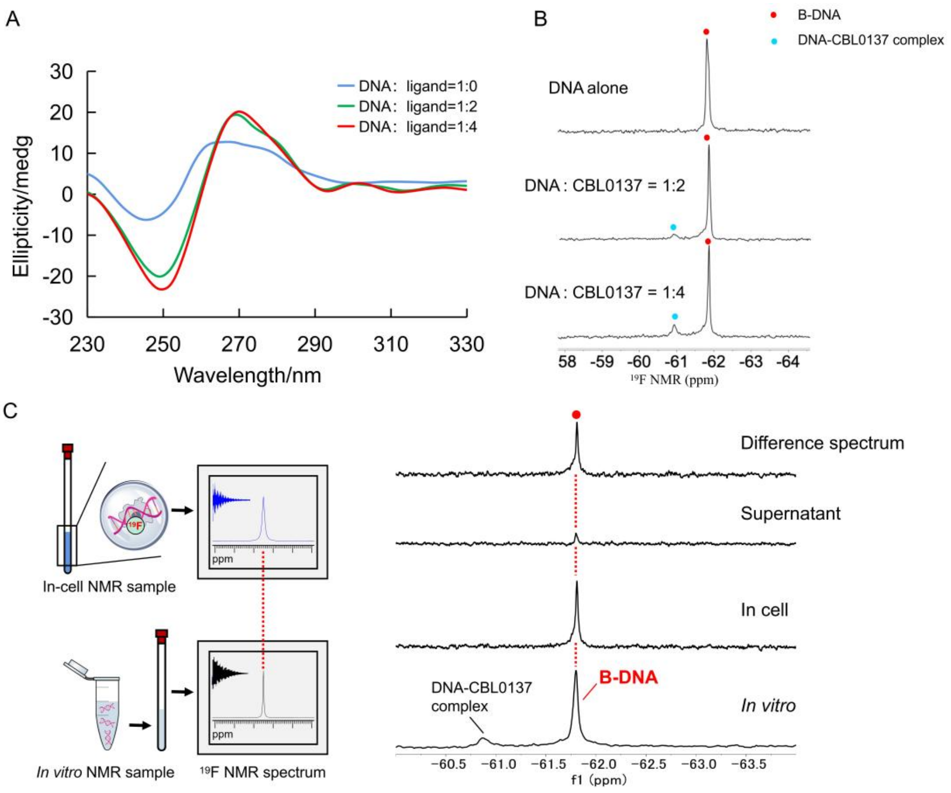

Figure S4. (A) CD spectra of 8-mer d(CTT^{8F}**G**CAAG)$_2$ with increasing concentrations of CBL0137 in 100 mM NaCl with 5 mM Na-PO4 buffer (pH 7.0). **(B)** ^{19}F NMR spectra of 8-mer d(CTT^{8F}**G**CAAG)$_2$ titrated with CBL0137 in 100 mM NaCl, 5 mM Na-PO$_4$ buffer (pH 7.0). The CBL0137-to-DNA molar ratio is indicated at the top. Red and sky-blue signals correspond to B-DNA and the DNA–ligand complex respectively. (**C**) ^{19}F NMR detection of the B-DNA in cells. Comparison of the in-cell spectrum with the *in vitro* reference enables reliable determination of intracellular structures. ^{19}F NMR spectra showing *in vitro* B-DNA, *in vitro* DNA–CBL0137 complex, and B-DNA in living HeLa cells, in the supernatant, and in the difference spectrum (cell minus supernatant).

Supplementary Table S1. ^{1}H chemical shift assignments of the Z-form DNA d(CGC**^{8F}G**CG)$_2$ at 20°C[a].

Residue	H5	H8/6	H1′	H2′	H2″	H3′	H4′	H5′	H5″	H1	H 2a/4a	H 2b/4b
C1	5.78	7.39	5.72	1.70	2.59	4.35	3.73	2.62	3.24	—	8.23	6.50
G2	—	7.84	6.31	2.82	2.52	4.87	3.96	4.35	4.28	13.22	8.37	6.80
C3	5.39	7.47	5.81	1.80	2.77	4.28	3.87	2.67	4.00	—	8.45	6.57
^{8F}G4	—	—	6.48	5.38	—	5.16	4.17	4.09	4.28	13.49	8.72	7.07
C5	5.28	7.41	5.79	1.73	2.67	4.28	3.92	2.79	3.79	—	8.45	6.58
G6	—	7.92	6.36	3.09	2.82	5.08	4.10	3.76	4.29	13.18	na	na

[a]na is "not applicable", representing ^{1}H chemical shifts could not be determined in NOESY spectrum.

Supplementary Table S2. Experimental constraints and calculation statistics of d(CGC**^{8F}G**CG)$_2$ and d(CGC**^{8F}G**CG)$_2$–CBL0137 complex.

Models	d(CGC**^{8F}G**CG)$_2$	d(CGC**^{8F}G**CG)$_2$–CBL0137 complex
Experimental distance constraints		
Total number	168	190
Intra-residue	108	84
Inter-residue	60	44
d(CGC**^{8F}G**CG)$_2$–CBL0137	—	62
Root Mean Square Deviation (RMSD) (Å)		
All bases	0.62 ± 0.09	0.60 ± 0.06
Backbone	0.86 ± 0.15	0.76 ± 0.08
All heavy atoms	0.75 ± 0.10	0.68 ± 0.09

Supplementary Table S3. Backbone and glycosidic torsion angles for 10 conformers representing NMR refined structure of Z-form d(CGC**^{8F}G**CG)$_2$[a].

Residue	α	β	γ	δ	ε	ζ	χ
C1	—	—	94.7 (3)	114.0 (5)	-86.6 (5)	71.4 (4)	-162.0 (6)
G2	78.1 (2)	-151.7 (4)	151.0 (3)	89.7 (2)	-135.0 (3)	-51.3 (1)	61.5 (2)
C3	-180.0 (2)	-124.0 (2)	64.5 (1)	130.1 (2)	-90.6 (2)	156.6 (4)	-143.8 (4)
^{8F}G4	-32.1 (1)	-83.0 (1)	-169.2 (4)	92.3 (3)	-146.9 (5)	-40.4 (1)	64.9 (2)
C5	-177.2 (6)	-128.0 (4)	46.4 (1)	122.2 (3)	-119.2 (2)	138.6 (4)	-158.7 (5)
G6	51.8 (2)	-124.6 (3)	162.4 (4)	104.7 (3)	—	—	79.0 (2)

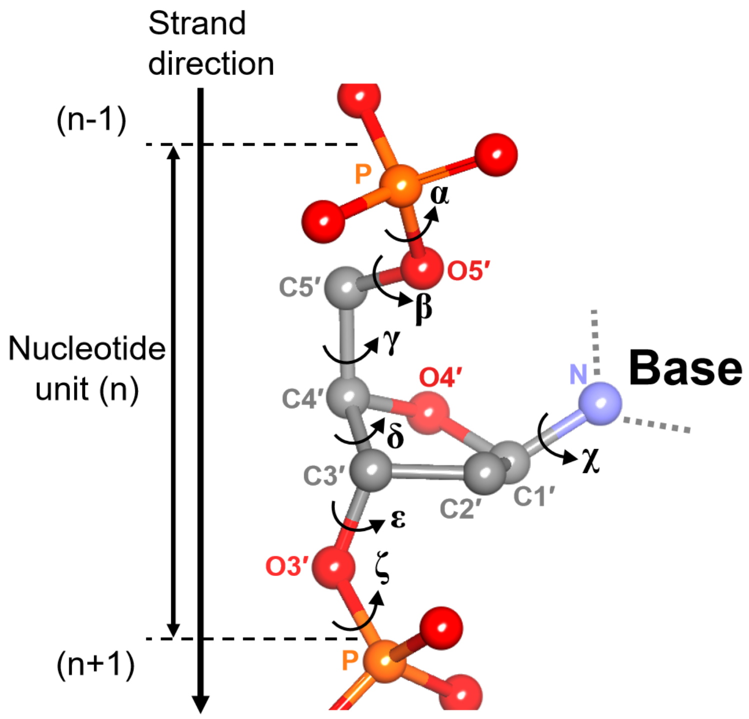

[a]A ball-stick model defines backbone and glycosidic torsion angles of one nucleotide unit within DNA double helix, following the direction from 5′- to 3′-end on DNA strand.

Supplementary Table S4. Selected helical parameters for 10 conformers representing NMR refined structure of Z-form d(CGC^{8F}**G**CG)$_2$[a].

Base steps	Shift [Å]	Slide [Å]	Rise [Å]	Tilt [°]	Roll [°]	Twist [°]
C1:G6—G2:C5	0.13 (0.1)	5.49 (0.8)	3.98 (0.5)	-2.39 (0.1)	10.89 (0.6)	-27.25 (1.5)
G2:C5—C3:^{8F}G4	0.05 (0.1)	-1.17 (0.2)	3.44 (0.2)	-3.35 (0.1)	-1.88 (0.1)	-43.76 (1.8)
C3:^{8F}G4—^{8F}G4:C3	0.01 (0.0)	4.40 (0.1)	3.07 (0.2)	0.81 (0.1)	3.57 (0.2)	-19.85 (0.9)
^{8F}G4:C3—C5:G2	0.05 (0.1)	-1.17 (0.1)	3.44 (0.3)	3.35 (0.5)	-1.88 (0.3)	-43.76 (1.4)
C5:G2—G6:C1	0.13 (0.1)	5.51 (0.6)	3.95 (0.4)	2.39 (0.3)	10.89 (1.2)	-27.25 (1.7)

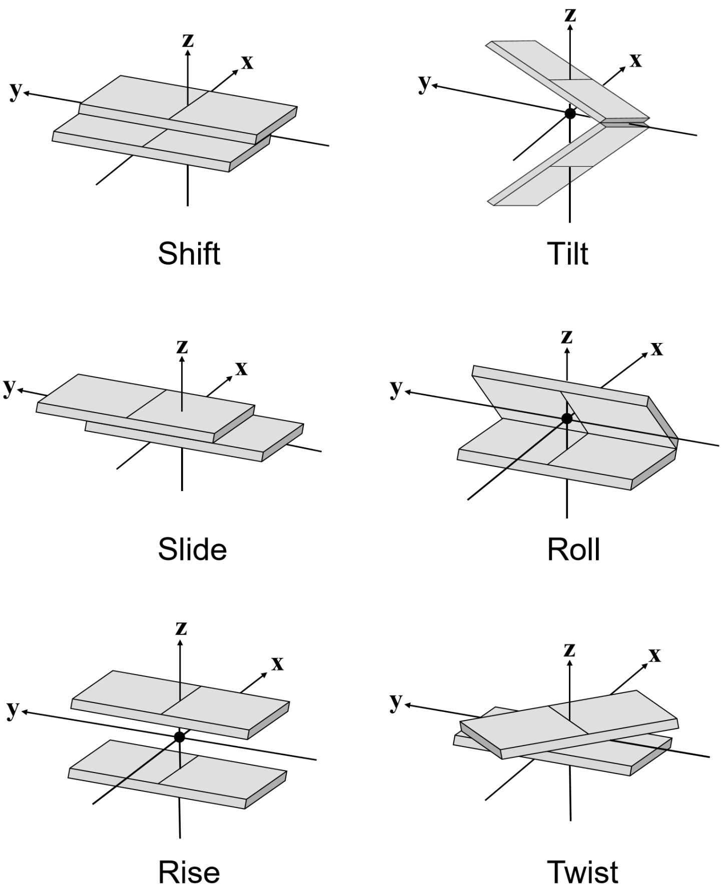

[a]Cartoon models indicate six helical parameters between neighbouring two base pairs in DNA duplex, including shift, slide, rise, tilt, roll and twist.

Supplementary Table S5. ^{1}H chemical shift assignments of ligand CBL0137 at 20°C.

Protons	1,8H	2,7H	4,5H	CH_2(9)	CH_2	CH (isopropyl)	CH_3 (isopropyl)	3,6CH_3
δ (ppm)	7.88	8.37	8.88	4.54	3.09	2.71	1.50	2.81

Supplementary Table S6. ^{1}H chemical shift assignments of ligand CBL0137 in Z-form d(CGC^{8F}**G**CG)$_2$–CBL0137 complex at 20°C.

Protons	1,8H	2,7H	4,5H	CH_2(9)	CH_2	CH (isopropyl)	CH_3 (isopropyl)	3,6CH_3
δ (ppm)	7.71	8.36	8.81	4.54	3.08	2.83	1.49	2.81

Supplementary Table S7. ^{1}H chemical shift assignments of Z-form d(CGC^{8F}**G**CG)$_2$ in Z-form d(CGC^{8F}**G**CG)$_2$–CBL0137 complex at 20°C[a].

Residue	H5	H8/6	H1′	H2′	H2″	H3′	H4′	H5′	H5″	H1	H 2a/4a	H 2b/4b
C1	5.74	7.47	5.72	1.84	2.65	4.20	3.75	2.61	3.28	—	8.35	6.58
G2	—	7.73	6.31	2.64	2.63	5.07	3.94	4.30	4.13	13.16	8.24	6.57
C3	5.27	7.44	5.82	1.85	2.68	4.80	4.30	2.88	3.73	—	8.29	6.58
^{8F}G4	—	—	6.37	5.47	—	5.23	4.35	na	4.33	13.50	8.74	7.08
C5	5.21	7.26	5.63	1.74	2.66	4.72	4.34	2.81	3.84	—	8.40	6.61
G6	—	7.99	6.43	3.28	2.85	5.16	4.28	4.31	4.24	12.34	na	na

[a]na is "not applicable", representing ^{1}H chemical shifts could not be determined in NOESY spectrum.

Supplementary Table S8. Intermolecular NOE-derived distance restraints between d(CGC[8F]**G**CG)$_2$ and CBL0137. NOE cross peak intensities were classified as strong (s, 1.8–3.0 Å), medium (m, 3.0–3.7 Å), and weak (w, 3.7–5.5 Å) by structure calculations.

CBL0137		1H	2H	$3CH_3$	4H	5H	$6CH_3$	7H	8H	CH_3 (isopropyl)
C1	H1′	w	w	w						
	H4′	w	s							
	H5″	w								
	H6		m							
G2	H2′		w							
	H5″									s
C3	H3′									s
	H5″									m
C5	H1′								w	
	H2″							w		
G6	H1					m				
	H1′						m			
	H2′							w		

Supplementary Table S9. Backbone and glycosidic torsion angles for 10 conformers representing NMR refined structure of d(CGC**^{8F}G**CG)$_2$–CBL0137 complex[a].

Residue	α	β	γ	δ	ε	ζ	χ
C1	—	—	92 (6)	81.8 (6)	-95.5 (8)	120.2 (6)	-144.1 (9)
G2	26.2 (2)	-138.5 (3)	-168.6 (2)	84.2 (2)	-110.1 (3)	-30.6 (2)	66.0 (2)
C3	-154.7 (1)	159.3 (2)	76.4 (1)	123.0 (2)	-97.5 (2)	147.2 (3)	-165.8 (4)
^{8F}G4	11.2 (1)	-103.5 (1)	-165.1 (3)	102.5 (3)	-145.1 (4)	-46.6 (1)	64.6 (2)
C5	154.5 (5)	-131.3 (3)	73.0 (1)	116.1 (2)	-65.7 (2)	69.5 (2)	-157.0 (4)
G6	64.7 (3)	-167.2 (5)	-150.0 (6)	79.0 (4)	—	—	62.8 (3)

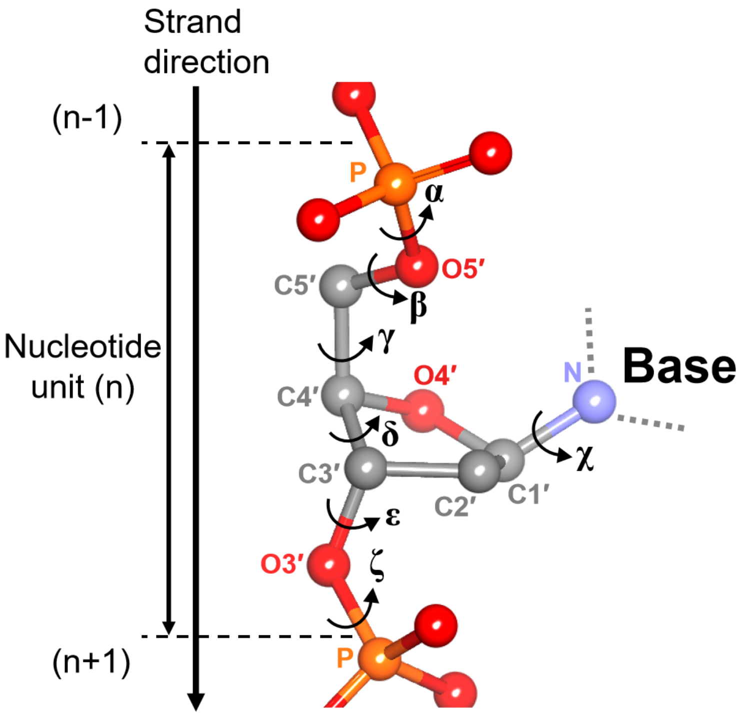

[a]A ball-stick model defines backbone and glycosidic torsion angles of one nucleotide unit within DNA double helix, following the direction from 5′- to 3′-end on DNA strand.

Supplementary Table S10. Selected helical parameters for 10 conformers representing NMR refined structure of d(CGC^{8F}**G**CG)$_2$–CBL0137 complex[a].

Base steps	Shift [Å]	Slide [Å]	Rise [Å]	Tilt [°]	Roll [°]	Twist [°]
C1:G6—G2:C5	0.11 (0.1)	3.55 (1.2)	6.87 (0.8)	-14.19 (0.3)	7.57 (0.8)	-14.70 (2.2)
G2:C5—C3:^{8F}G4	0.11 (0.1)	-1.63 (0.2)	3.21 (0.1)	-1.74 (0.1)	2.68 (0.2)	-43.65 (0.6)
C3:^{8F}G4—^{8F}G4:C3	-0.26 (0.1)	4.35 (0.1)	3.18 (0.1)	-1.61 (0.1)	7.13 (0.1)	-21.45 (0.2)
^{8F}G4:C3—C5:G2	0.10 (0.1)	-1.50 (0.1)	3.20 (0.2)	-1.77 (0.1)	2.66 (0.2)	-43.62 (0.7)
C5:G2—G6:C1	0.10 (0.1)	3.47 (0.7)	6.92 (0.8)	-14.08 (1.2)	7.48 (1.9)	-13.04 (2.0)

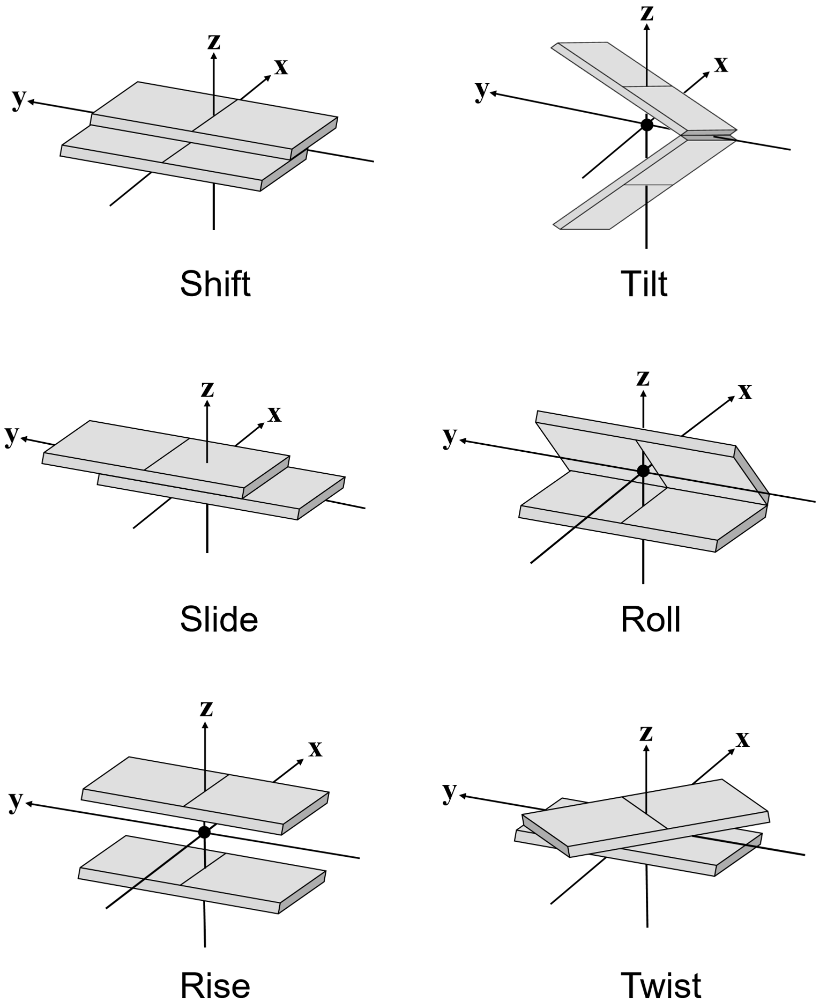

[a]Cartoon models indicate six helical parameters between neighbouring two base pairs in DNA duplex, including shift, slide, rise, tilt, roll and twist.

Supplementary Table S11. Structural parameters comparison between B-DNA and Z-DNA[1–3].

Parameters	B-DNA	Z-DNA
Helical handedness	Right	Left
Residues per turn	10.0	12.0
Diameter	20.0 Å	18.0 Å
Helical slide per base pair	0.5 Å	3.5 Å
Helical rise per base pair	3.4 Å	3.6 Å
Major groove	12.0 Å	—
Minor groove	5.8 Å	7.7 Å
Sugar pucker	C2′-endo	C2′-endo (dC)<br>C3′-endo (dG)
Glycosidic bond conformation	*Anti*-form	*Anti*-form (dC)<br>*Syn*-form (dG)

Supplementary Table S12. DNA molecular modeling energies in CHARMM36 force field[a].

Molecular modelings	B-DNA	Z-DNA	Z-DNA–CBL0137 complex	Difference (B–Z)	Difference (Z–complex)	Difference (B–complex)
8-mer: d(CGCACGCG)/ d(CGCGTGCG)	-5725.09	-5706.69	-5727.29	+18.49	-20.60	-2.20
16-mer:d(CGCACGCGCGCACGCG)/ d(CGCGTGCGCGCGTGCG)	-8605.73	-8590.96	-8632.03	+14.77	-41.07	-26.30

[a] All values are in kcal/mol.

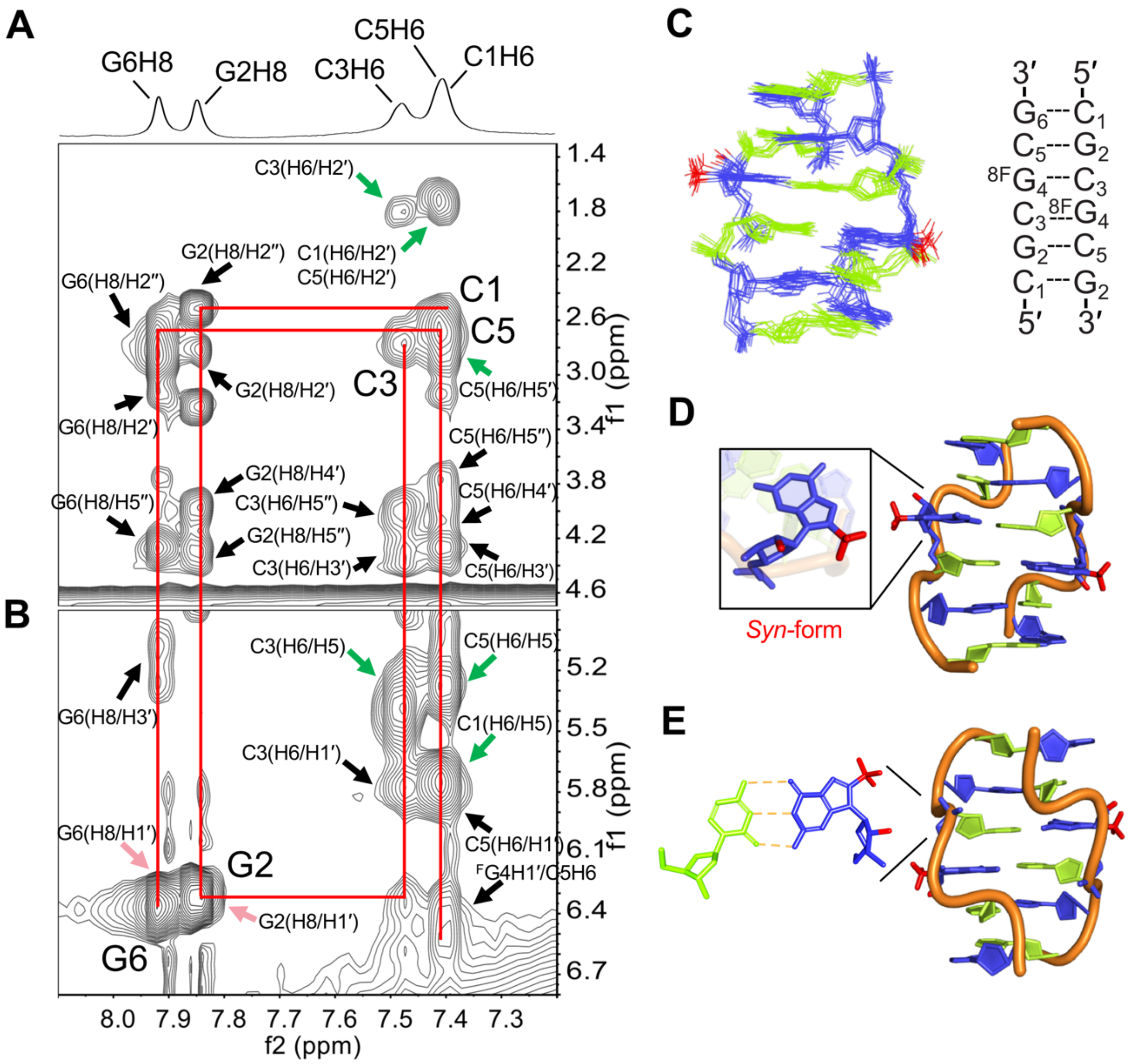

Figure S5. Structural determination of Z-form DNA d(CGC^{8F}**G**CG)$_2$. (**A, B**) 2D-NOESY spectra showing connectivity pathways (red lines) [C1(H6/H2″)–G2(H8/H1′)–C3(H6/H2″), C5(H6/H2″)–G6(H8/H1′)], characteristic of left-handed Z helices. Black arrows indicate inter- and intranucleotide anomeric–aromatic proton interactions (C1H6, G2H8, C3H6, C5H6, C6H8). Green arrows highlight upfield-shifted signals: (**A**) cytidine H2′ resonances at intra-residue H6/H2′ cross peaks at 1.70 (C1H2′), 1.80 (C3H2′), and 1.73 ppm (C5H2′) ; (**B**) cytosine H5 signals at 5.78 (C1H5), 5.39 (C3H5), and 5.28 ppm (C5H5). Strong G2(H8/H1′) and G6(H8/H1′) cross peaks confirm syn conformations of dG residues in Z-DNA. (**C**) Molecular dynamics ensemble of 10 conformers (line representation) viewed from the major groove. (**D**) Ribbon model of Z-DNA viewed from the major groove, showing the characteristic left-handed zig-zag phosphate backbone; ^{8F}**G4** is expanded to highlight its *syn* conformation. (**E**) Ribbon model viewed from the minor groove. Green, blue, and red represent dC, dG, and CF_3/2′F modifications, respectively. The C:^{8F}**G4** base pair is expanded to show three hydrogen bonds (orange dashed lines).

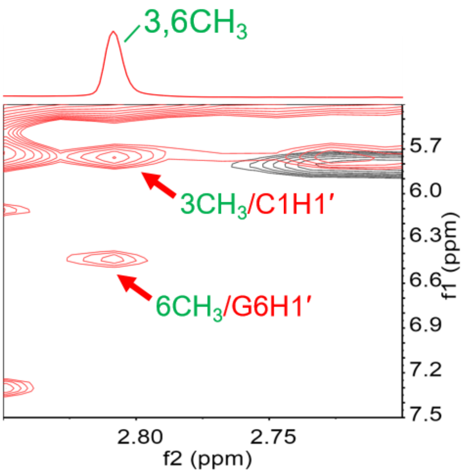

Figure S6. The overlay of NOE spectra. The NOEs in red coming from the complex of CBL0137 and DNA. The signals in black from Z-form DNA alone as control. The NOEs of 3CH3/C1H1′ and 6CH3/G6H1′ show interaction between Z-DNA and ligand CBL0137 labeled by red arrows. Red labels represent atoms coming from DNA and green labels represent atoms coming from ligand.

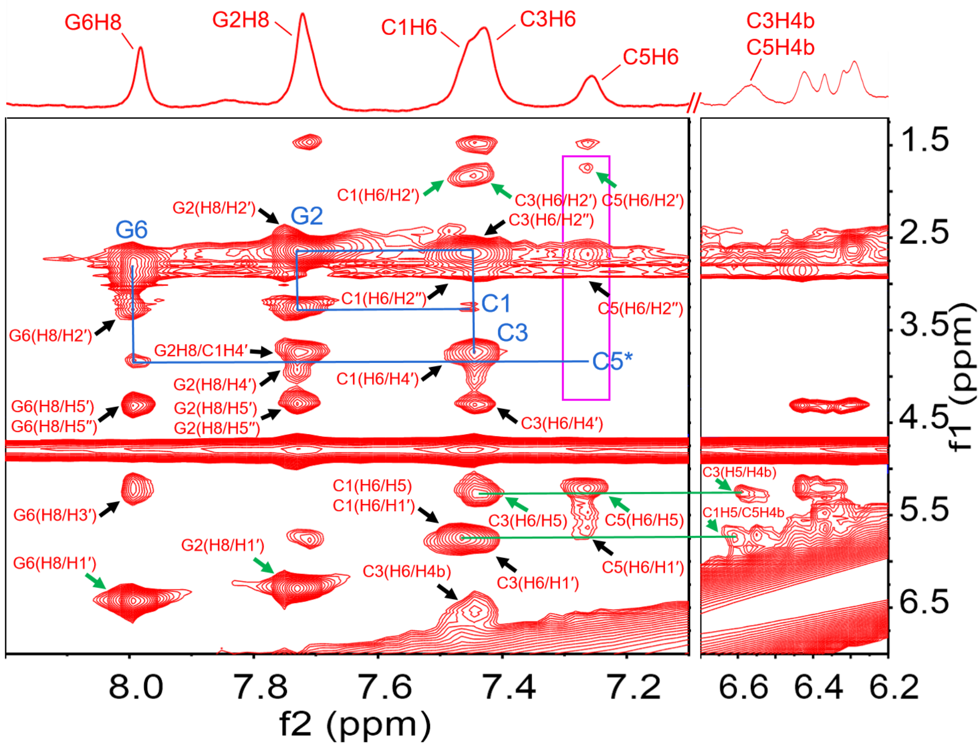

Figure S7. Structural determination of Z-form DNA in CBL0137–DNA complex by NOESY spectroscopy. Wide-ranging connectivity paths (blue lines) [C1(H6/H5″)-G2(H8/H2″)-C3(H6/H5″), C5(H6/H5″)-G6(H8/H2″)] in the DNA strand of left-handed Z helices. Additionally, intranucleotide anomeric-aromatic proton interactions were assigned from C1H6, G2H8, C3H6, C5H6 and C6H8 according to ^{1}D spectrum respectively, labeling by black arrows. Strong intra-residue cross peaks of H6/H2′ show these H2′ atoms with upshift to 1.84 (C1H2′), 1.85 (C3H2′) and 1.74 (C5H2′) ppm remarked by green arrows. The cross peaks of C3(H6/H5) and C5(H6/H5) indicate the H5 of cytosine with upfield shift to 5.27 (C3H5) and 5.21 (C5H5) ppm remarked by green arrows. Strong signals from G2(H8/H1′) and G6(H8/H1′) represent dG residues adopting *syn* conformation in Z-form DNA, labeled by green arrows. Z-DNA structure-specific cross peaks are observed between the C5 amino proton and C1H5 as well as C3 amino proton and C3H5 from inter-strand interactions at CpG steps with green arrows. These signals are linked to C1(H6/H5) and C3(H6/H5) by green lines. A pink rectangle marks the C5H6 having much weak NOEs to its ribose protons induced by shielding effect of ligand binding.

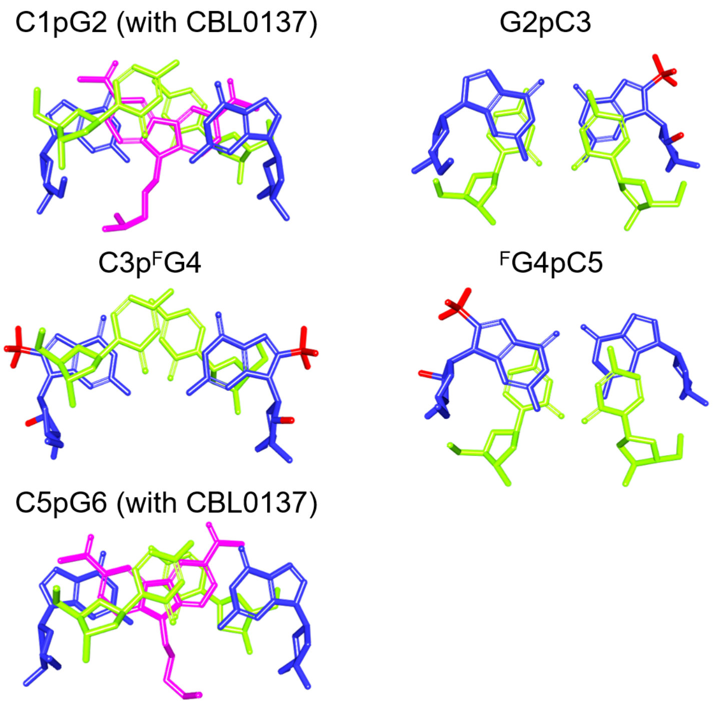

Figure S8. Stacking pattern within the CpG and GpC steps of Z-form DNA in CBL0137–DNA complex as viewed along the helix z-axis. The CpG steps show clear inter-strand stacking as shown by C1pG2, C3p^{8F}G4 and C5pG6, while GpC steps indicate no inter-strand overlay in Z-DNA, in which ligand inserting into C1pG2 and C5pG6 are indicated.

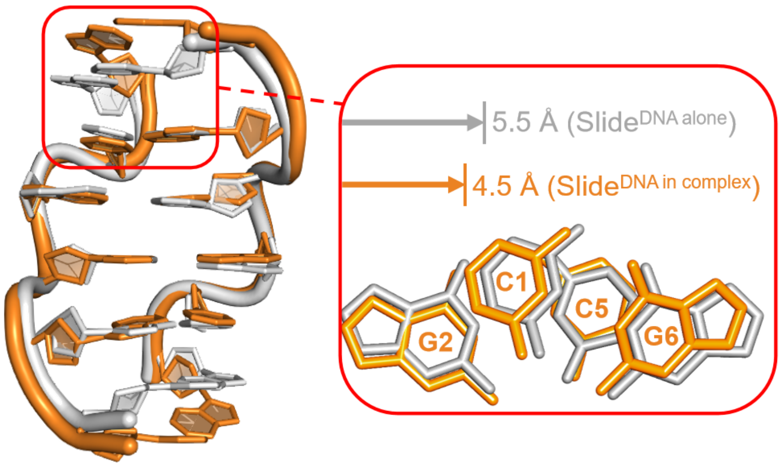

Figure S9. Comparison of Z-form DNA (orange) in CBL0137–DNA complex with Z-form DNA alone (gray), in which ligand is removed for convenience of observing DNA structure. The Z-DNA shown by cartoon presentation at major groove. The region with red label was expanded to indicate CpG steps including C1pG2 and C5pG6, adopting a lower slide when Z-form DNA in complex.

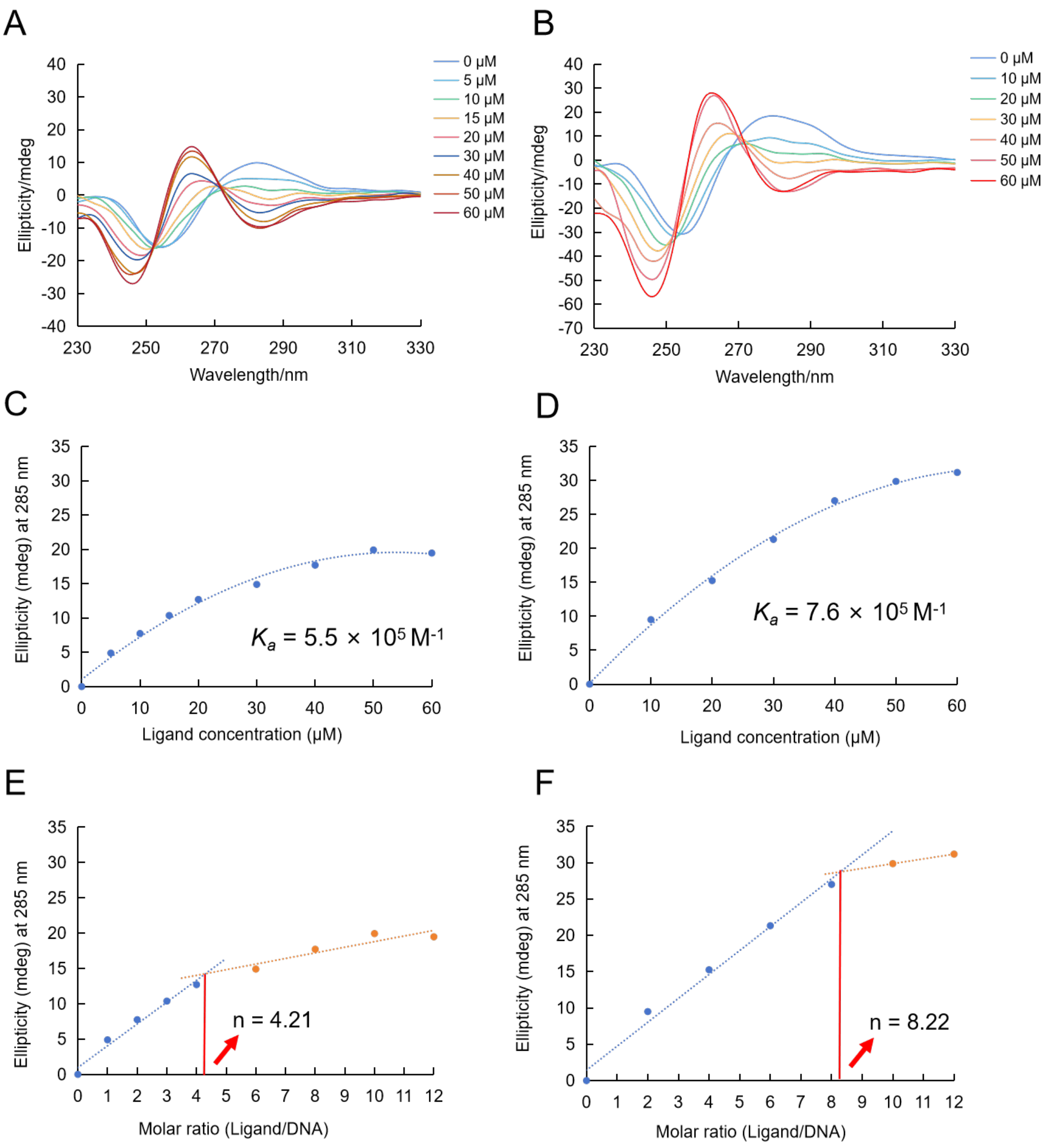

Figure S10. Biophysical studies of CBL0137 bounding to DNA. (A) CD spectra of the native 8-mer duplex d(CGCACGCG)/d(CGCGTGCG) upon titration with increasing concentrations of CBL0137 in 5 mM Na–PO_4 buffer (pH 7.0) containing 20 mM NaCl. (B) CD spectra of the native 16-mer d(CGCACGCGCGCACGCG)/d(CGCGTGCGCGCGTGCG) upon titration with increasing concentrations of CBL0137 in 5 mM Na–PO_4 buffer (pH 7.0). Titration data of CBL0137 and DNA derived by monitoring the θ value at 285 nm of CD. The solid lines are the theoretical fit of the data. The binding constants K_a of CBL0137 to 8-mer (C) and 16-mer DNA (D) are indicated. (E) Job plot of CD cotton effect monitored at 285 nm of 8-mer DNA. (F) Job plot of CD cotton effect monitored at 285 nm of 16-mer DNA.

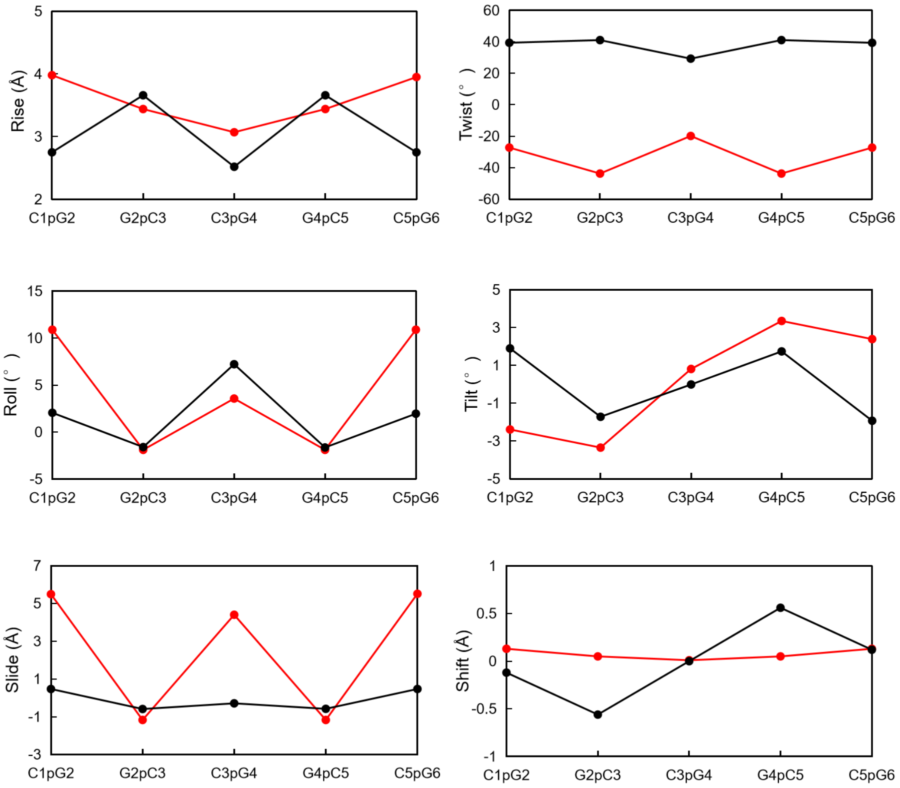

Figure S11. Comparison of the structural parameters extracted from Z-form d(CGC^{8F}GCG)$_2$ (red label) and a B-form d(CGCGCG)$_2$ (black label, PDB:1UQG)[4].

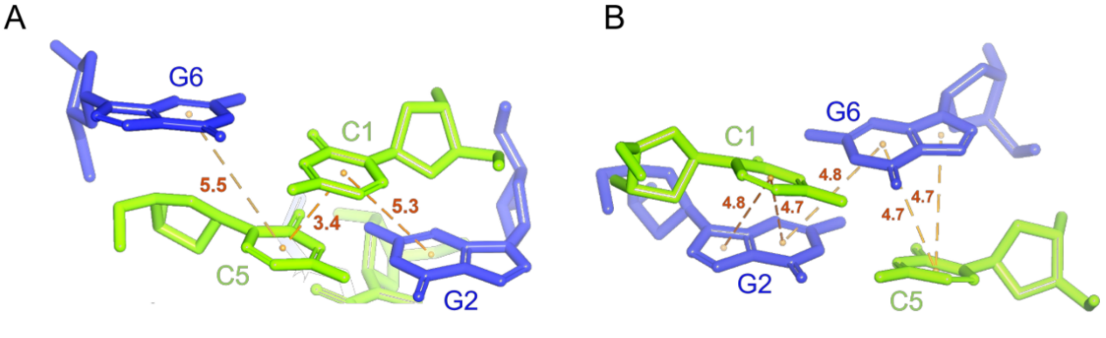

Figure S12. Base pair π-π stacking interactions between C1:G6 and G2:C5 at CpG step in comparison of Z-DNA (A) and B-DNA (B), in which hydrophobic interactions were indicated by dashed lines and their distances were shown.

References

1. Cui S, Wang Y, Chen G. Disturbance of DNA conformation by the binding of testosterone-based platinum drugs via groove-face and intercalative interactions: a molecular dynamics simulation study. BMC Struct Biol. 2013;13(1):4.
2. Harteis S, Schneider S. Making the bend: DNA tertiary structure and protein-DNA interactions. Int J Mol Sci. 2014;15(7):12335-12363.
3. Krall JB, Nichols PJ, Henen MA, Vicens Q, Vögeli B. Structure and formation of Z-DNA and Z-RNA. Molecules. 2023;28(2):843.
4. Lam SL, Au-Yeung SC. Sequence-specific local structural variations in solution structures of d $(CGXX'CG)_2$ and d $(CAXX'TG)_2$ self-complementary deoxyribonucleic acids. J Mol Biol. 1997;266(4):745-760.
